# An Alteration in ELMOD3, an Arl2 GTPase-Activating Protein, Is Associated with Hearing Impairment in Humans

**DOI:** 10.1371/journal.pgen.1003774

**Published:** 2013-09-05

**Authors:** Thomas J. Jaworek, Elodie M. Richard, Anna A. Ivanova, Arnaud P. J. Giese, Daniel I. Choo, Shaheen N. Khan, Sheikh Riazuddin, Richard A. Kahn, Saima Riazuddin

**Affiliations:** 1 Division of Pediatric Ophthalmology, Cincinnati Children's Hospital Medical Center, Cincinnati, Ohio, United States of America; 2 Division of Pediatric Otolaryngology Head & Neck Surgery, Cincinnati Children's Hospital Research Foundation, and Department of Otolaryngology, College of Medicine, University of Cincinnati, Cincinnati, Ohio, United States of America; 3 Department of Biochemistry, Emory University School of Medicine, Atlanta, Georgia, United States of America; 4 National Center for Excellence in Molecular Biology, University of the Punjab, Lahore, Pakistan; 5 Jinnah Hospital Complex, Allama Iqbal Medical College, University of Health Sciences, Lahore, Pakistan; Stanford University School of Medicine, United States of America

## Abstract

Exome sequencing coupled with homozygosity mapping was used to identify a transition mutation (c.794T>C; p.Leu265Ser) in *ELMOD3* at the *DFNB88* locus that is associated with nonsyndromic deafness in a large Pakistani family, PKDF468. The affected individuals of this family exhibited pre-lingual, severe-to-profound degrees of mixed hearing loss. ELMOD3 belongs to the engulfment and cell motility (ELMO) family, which consists of six paralogs in mammals. Several members of the ELMO family have been shown to regulate a subset of GTPases within the Ras superfamily. However, ELMOD3 is a largely uncharacterized protein that has no previously known biochemical activities. We found that in rodents, within the sensory epithelia of the inner ear, ELMOD3 appears most pronounced in the stereocilia of cochlear hair cells. Fluorescently tagged ELMOD3 co-localized with the actin cytoskeleton in MDCK cells and actin-based microvilli of LLC-PK1-CL4 epithelial cells. The p.Leu265Ser mutation in the ELMO domain impaired each of these activities. Super-resolution imaging revealed instances of close association of ELMOD3 with actin at the plasma membrane of MDCK cells. Furthermore, recombinant human GST-ELMOD3 exhibited GTPase activating protein (GAP) activity against the Arl2 GTPase, which was completely abolished by the p.Leu265Ser mutation. Collectively, our data provide the first insights into the expression and biochemical properties of ELMOD3 and highlight its functional links to sound perception and actin cytoskeleton.

## Author Summary

Autosomal recessive nonsyndromic hearing loss is a genetically heterogeneous disorder. Here, we report a severe-to-profound mixed hearing loss locus, *DFNB88* on chromosome 2p12-p11.2. Exome enrichment followed by massive parallel sequencing revealed a c.794T>C transition mutation in *ELMOD3* that segregated with DFNB88-associated hearing loss in a large Pakistani family. This transition mutation is predicted to substitute a highly invariant leucine residue with serine (p.Leu265Ser) in the engulfment and cell motility (ELMO) domain of the protein. No biological activity has been described previously for the ELMOD3 protein. We investigated the biochemical properties and ELMOD3 expression to gain mechanistic insights into the function of ELMOD3 in the inner ear. In rodent inner ears, ELMOD3 immunoreactivity was observed in the cochlear and vestibular hair cells and supporting cells. However, ELMOD3 appears most pronounced in the stereocilia of cochlear hair cells. *Ex vivo*, ELMOD3 is associated with actin-based structures, and this link is impaired by the *DFNB88* mutation. ELMOD3 exhibited GAP activity against Arl2, a small GTPase, providing a potential functional link between Arf family signaling and stereocilia actin-based cytoskeletal architecture. Our study provides new insights into the molecules that are necessary for the development and/or function of inner ear sensory cells.

## Introduction

Many molecular components that are necessary for the development and maintenance of hearing have been discovered by identifying the genes that underlie hearing impairment in humans and mice [1]–[4]. Hearing requires the precise and efficient functioning of intricately structured mechanosensory hair cells and supporting cells in the inner ear [3]. One of the key structures in the mechanotransduction process is the hair cell stereocilium. Protruding from the apical surface of the hair cells, stereocilia are organized in three rows of decreasing height in a staircase pattern. Each stereocilium is composed of an actin core that contains cross-linked and bundled γ- and β-actin microfilaments that are uniformly polarized, with the barbed (positive) ends localized at the tip. At the tapered end of the stereocilium, the actin filaments form a rootlet that has been proposed to anchor the structure in the actin-rich meshwork of the cuticular plate [5]. Interestingly, among the identified hearing loss-associated genes, nineteen encode proteins that interact with actin [6], [7]. Numerous studies have demonstrated that actin cytoskeleton-associated proteins are involved in the development, maintenance and stabilization of the stereocilia (for review, see [6]).

Continuous depolymerization of actin filaments at the base and polymerization at the barbed end, termed treadmilling, is thought to be critical to the maintenance of the length of stereocilia [8], [9]. However, a recent study demonstrated a rapid turnover of the actin filaments only at the tip of the stereocilia, without a treadmilling process [10], emphasizing the specific role of proteins at the stereocilia tip in the regulation of actin filaments. Regardless of the precise site, it is quite clear that the proper regulation of actin dynamics is critical to the generation and maintenance of stereocilia as sensory structures.

The Rho/Rac/Cdc42 family of GTPases is well known as a regulator of actin. Rho and Rac in the inner ear are involved in the morphogenesis and growth of the otocyst [11], [12]. The depletion of Rac1 or both Rac1 and Rac3 in the murine inner ear leads to a shorter cochlear duct with an abnormal sensory epithelium. Rac may participate in cell adhesion, proliferation, and movements during otic development [11], [12]. Several studies have suggested that the activation/inhibition of Rho pathways control the actin depolymerization rate in the outer hair cells [13], [14]. Although best known for their roles in the regulation of membrane traffic, there is growing evidence that GTPases in the Arf family can also act via changes in actin. [15].

Here, we report the identification of a new deafness gene, which encodes an ELMO/CED 12 domain containing protein, *ELMOD3*. Our biochemical studies demonstrated that ELMOD3 possesses GAP activity against a small GTPase in the Arf family, Arl2, providing a functional link between Arf family signaling pathways and stereocilia actin-based cytoskeletal architecture. GAPs are regulators and effectors of the Ras superfamily of GTPases, which are increasingly recognized as providing specificity as well as temporal and spatial regulation to GTPase signaling [16]. Thus, we believe that the identification of ELMOD3 role in the inner ear provides new insights into signaling processes that are important to hearing in humans.

## Results

### Clinical findings

**Figure 1:**
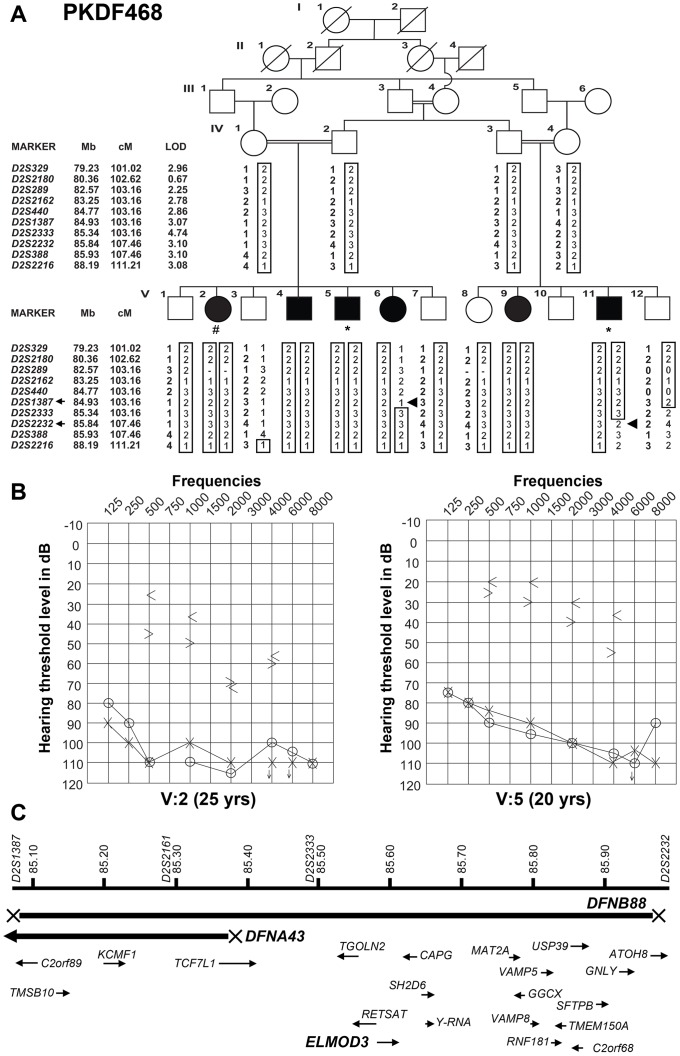
Hearing loss segregating in family PKDF468 is associated with a missense ELMOD3 allele. (A) Pedigree of the family in which the *DFNB88* locus was mapped. The filled symbols represent affected individuals, and a double horizontal line connecting parents represents a consanguineous marriage. The alleles forming the risk haplotypes are boxed. The short tandem repeat (STR) markers, their relative map positions (Mb) according to UCSC Genome Bioinformatics Build GRCh37 (hg19), and their genetic positions (cM) based on the Marshfield genetic map are shown next to the pedigree. Haplotype analysis revealed a linkage region of 4.3 cM (0.91 Mb) that was delimited by proximal meiotic recombination in individual V:6 (arrowhead) at marker *D2S1387* (103.16 cM; arrow) and distal recombination at marker *D2S2232* (107.46 cM; arrow) in individual V:11 (arrowhead). (B) Pure-tone audiograms for family PKDF468 V:2 (25-year-old female) and V:5 (20-year-old male). The symbols ‘o’ and ‘x’ denote air conduction pure-tone thresholds, and the ‘<’ and ‘>’ symbols denote bone conduction thresholds in the right and the left ears, respectively. (C) The linkage interval that defines the *DFNB88* locus for family PKDF468 partially overlaps with *DFNA43*. The relative locations and orientations of the genes and mRNAs are indicated with arrows. Individual V:2 DNA sample was used for exome sequencing, while individuals V:5 and V:11 samples were used for Sanger sequencing of coding, non-coding, and flanking sequences of the exon-intron boundaries of the known candidate genes in *DFNB88* linkage interval.

Family PKDF468 (Figure 1A) was recruited after obtaining Institutional Review Board approval and written informed consent. The family history revealed that the onset of hearing loss was pre-lingual, with no clear vestibular impairment among the deaf individuals. Pure-tone bone and air-conduction audiometry revealed severe-to-profound mixed (conductive and sensorineural) hearing loss in the affected individuals of family PKDF468 (Figure 1B). Individual V:2 exhibited severe-to-profound mixed hearing loss, with bone conduction thresholds for the right ear displaying a mild downward slope to the severe hearing loss range. The left ear displayed slightly better bone conduction thresholds with normal values for lower frequencies and a downward slope to severe hearing loss at higher frequencies (Figure 1B). The audiograms of individual V:5 revealed bilateral severe-to-profound mixed hearing loss, with a large conductive component in both ears. The bone conduction thresholds exhibited a mild downward slope to moderately severe hearing loss for the right ear and were slightly better on the left, for which the thresholds ranged from borderline normal to moderate hearing loss ranges (Figure 1B). The clinical evaluation revealed no clear signs of skin, renal, or retinal abnormalities.

To determine the temporal bone malformation, we performed computed tomography (CT) scans of two affected (V:2 and V:11) along with a normal hearing sibling (V:7). CT scan of individual V:2 revealed all three semicircular and internal auditory canals were intact on both sides. The middle ear and mastoid appeared well-aerated bilaterally. Imaging of individual V:11 demonstrated a slightly narrow appearing internal auditory canal on the right side only. The mastoid air cells and middle ear cleft were well-aerated bilaterally. The external auditory canal appeared normal as well for both affected individuals.

### Deafness in PKDF468 is linked with *DFNB88* locus

We initially observed that deafness in family PKDF468 did not co-segregate with short tandem repeat (STR) markers for 74 of the reported recessive nonsyndromic deafness loci (data not shown). We therefore performed a genome-wide linkage analysis and observed that the deafness phenotype of family PKDF468 exhibited significant evidence of linkage to STR markers on chromosome 2p12-p11.2 (Figure 1A). Additional STRs on 2p were genotyped, and haplotype analysis revealed a 0.91 Mb linkage interval that was delimited by the markers *D2S1387* and *D2S2232* (Figure 1A). Under a recessive model of inheritance, with a disease allele frequency of 0.001 and full penetrance, a maximum two-point LOD [17] score of 4.74 (θ = 0) was obtained for the marker *D2S2333*. These results define and delimit *DFNB88* [Human Genome Nomenclature Committee (HGNC) approved locus symbol], a novel recessive deafness locus on chromosome 2p11.2.

### Exome sequencing revealed a mutation in *ELMOD3* at the *DFNB88* locus

The *DFNB88* locus partially overlaps with the dominant deafness locus *DFNA43* (Figure 1C) [18]. Four known candidate genes were identified within the *DFNB88/DFNA43* overlapping linkage region (Figure 1C). However, Sanger sequencing of these genes did not reveal any pathogenic variants. Approximately 85% of the disease-causing mutations in Mendelian disorders reside in coding regions or in exon-intron canonical splice junctions [19]. We therefore performed exome sequencing of an affected individual from family PKDF468. The sample was enriched using the NimbleGen SeqCap EZ Exome Library v2.0 (Roche Diagnostics; San Francisco, CA), and 100 bp, paired-end sequencing was performed on the Illumina HiSeq 2000 platform (Illumina). An average of 78.94% of bases were sequenced with 20× coverage within the targeted regions. This yielded a total of 64,863 single-nucleotide variants, of which 1,928 were not found in the dbSNP133 database (Table S1). Based on the recessive mode of inheritance evident in the pedigree, we analyzed genes with homozygous changes and potential compound heterozygous changes. Additionally, we removed all of the variants that were present in six ethnically matched control samples (Table S1). No mutation segregating with hearing loss in family PKDF468 was identified in any of the known deafness-causing genes (Table S1).

**Figure 2:**
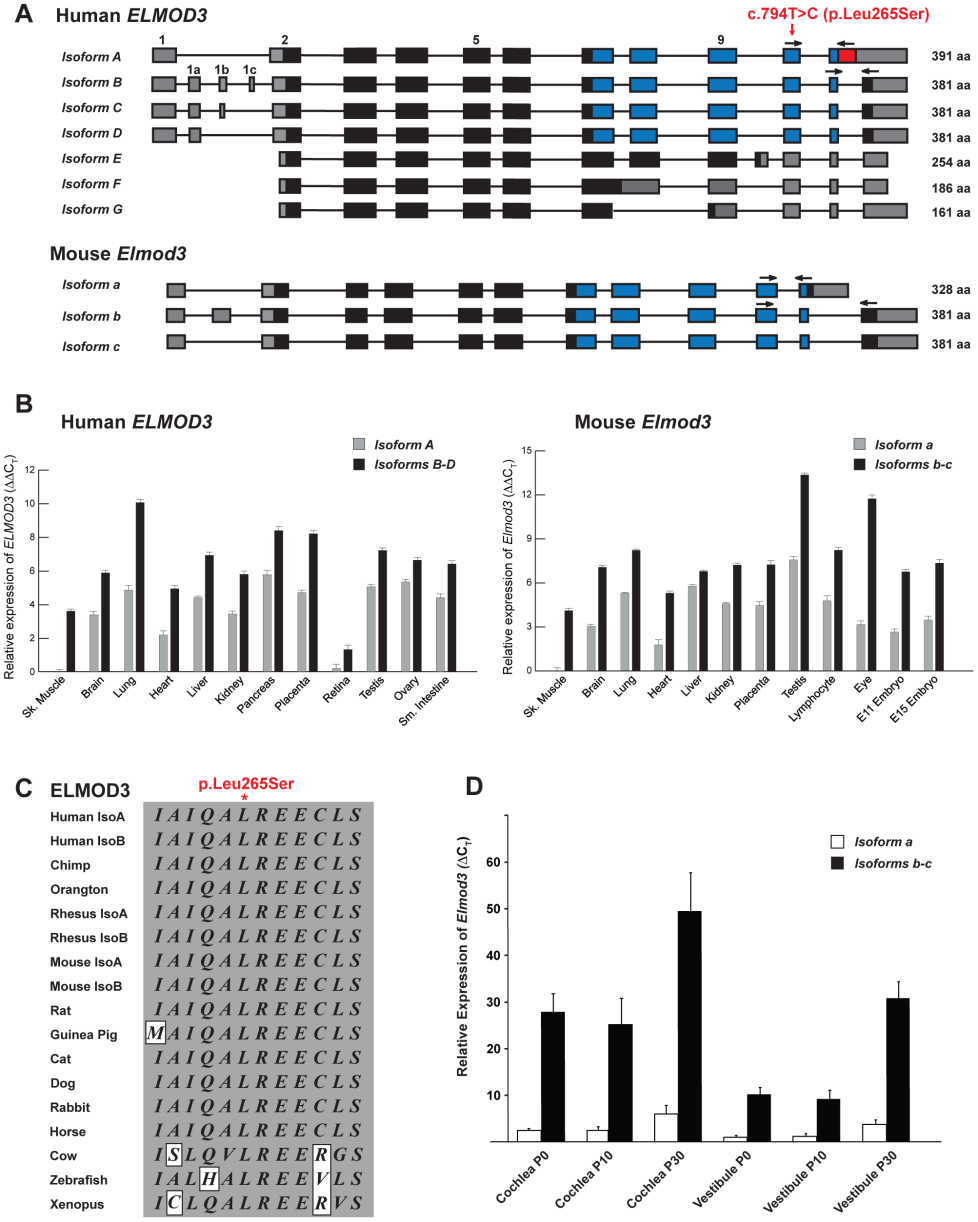
The transcripts and expression profiles of the genes that encode ELMOD3 in humans and mice. (A) Alternative splicing leads to seven isoforms of human *ELMOD3*. Non-coding segments, sequences encoding ELMO domain and other coding regions of exons are denoted by gray, blue and black boxes, respectively. Also depicted is the mutation that was identified in the DFNB88 family (red arrow). The numbering of the position of mutation c.794T>C (p.Leu265Ser) is based on accession number NM_032213.4. Mice have only three *Elmod3* alternative transcripts. Primers used for real-time quantitative PCR analyses are represented with black arrows. (B) Real-time quantitative PCR analysis of human and mouse *ELMOD3/Elmod3* isoforms *A/a* and *B–E/b–c*. (C) The leucine residue at amino acid position 265 (accession number NP_115589) is completely conserved across a wide variety of species. Identical residues are boxed in gray. (D) Real-time quantitative RT-PCR analysis of *Elmod3* isoforms *a* and *b–c* in C57BL/6J mouse cochlear and vestibular tissues at three different ages (P0, P10 and P30). C_T_ indicates the observed threshold number of PCR cycles that was required for the detection of the amplification product; the relative expression level is the calculated difference in C_T_ between the *Elmod3* and that of an internal control standard (*Gapdh*), which was measured in the same sample.

We identified one homozygous transition mutation, c.794T>C (p.Leu265Ser), in *ELMOD3* (Figure S1) on chromosome 2p11.3 (Figure 1C) that segregated with *DFNB88*-linked deafness (Tables S1 and S2). The c.794T>C change was not present near the canonical splice junctions and was not predicted to create any aberrant splice site. However, to confirm that c.794T>C did not affect splicing of *ELMOD3* transcripts, we generated cDNA libraries using the total RNA extracted from the white blood cells of two affected and one normal hearing individual. Sanger sequencing of sub-cloned PCR products, amplified using primers in either exons 9 and 11 or in exons 9 and 12 (Figure S2), did not reveal any aberrant splicing product in affected individuals. Thus, the likely pathogenic affect of the c.794T>C change is substitution of a highly conserved leucine residue at amino acid position 265 of the human ELMOD3 protein with serine (Figure 2C). No carrier of c.794T>C was identified among 524 ethnically matched control chromosomes, in the 1000 Genome database or in the 6500 individuals who are listed in the NHLBI-ESP variant database (http://evs.gs.washington.edu/EVS/). Moreover, Polyphen-2 [20], SNPs3D [21], MutationTaster [22], PMut [23], and SIFT [24] predicted that the *ELMOD3* mutation would be deleterious (Table S3).

To further confirm that the p.Leu265Ser allele of *ELMOD3* is the only mutation that was associated with hearing loss at the *DFNB88* locus, we sequenced the coding, non-coding, and approximately 75 bp flanking sequences of the exon-intron boundaries of all the known candidate genes present within the linkage region in two affected individuals of family PKDF468 (Figure 1C). No other potentially pathogenic mutation was identified in the affected individuals of family PKDF468. Although, *ELMOD3* is located outside the reported linkage interval of *DFNA43* (Figure 1C) [18], nevertheless we sequenced DNA samples of two affected individuals from the original DFNA43 family and no mutation was found.

### *ELMOD3*/*Elmod3* are expressed ubiquitously

We next examined the gene structure and expression of *ELMOD3*. Seven alternatively spliced isoforms of human *ELMOD3* were identified (Figure 2A). Isoform A (reference sequence NM_ 032213.4) has a translation initiation codon (AUG) in exon 2, ten coding exons that encode a polypeptide of 391 residues (Figure S1B). Exons 7 to 11 encode the engulfment and cell motility (ELMO or CED12) domain, which consists of 164 amino acid residues (Figure S1B; blue box). *ELMOD3* isoforms *B* to *D* include alternatively spliced exons in the 5′ untranslated region (UTR) but harbor the same coding exons and encode identical 381 residue polypeptides that differ from isoform A only at their carboxy termini (Figures 2A and S1B). The human ELMOD3 isoforms, A and B, share 87% identity, with all the differences clustered near the C-terminus. Isoforms E, F, and G do not encode the full-length ELMO domain due to alternate splicing of exons in the carboxy terminus (Figure 2A). The c.794T>C transition mutation is predicted to result in the substitution of serine for a highly conserved leucine in all of the ELMO domain-containing isoforms of ELMOD3 (Figures 2A and 2C). In comparison to the human sequence, mouse *Elmod3* includes only three known alternatively spliced transcripts (Figure 2A). RT-PCR and real-time quantitative PCR analysis of multiple human and mouse tissue cDNAs (Tables S4 and S5) revealed the ubiquitous expression of isoforms *A/a* and *B–D/b–c* (Figures S3 and 2B). We also assayed the relative mRNA expression of murine *Elmod3* isoforms *a* and *b–c* with real-time quantitative RT-PCR of RNA that was extracted from cochlear and vestibular inner ear tissues from postnatal day 0 (P0), P10, and P30 C57BL/6J mice (Figure 2D). The expression of *Elmod3* isoform *b–c* was several-fold higher than isoform *a*, in both cochlear and vestibular tissues at all of the time points examined (Figure 2D). Therefore, we focused on the ELMOD3 isoform B for the subsequent biochemical and cellular studies. The mouse ELMOD3 protein is 70% and 80% identical to the A and B isoforms of human ELMOD3, respectively, and again the differences are greatest at the C-terminus, although single amino acid changes are scattered throughout the alignments.

### ELMOD3 is localized to the mechanosensory stereocilia of hair cells in rodent organ of Corti

**Figure 3:**
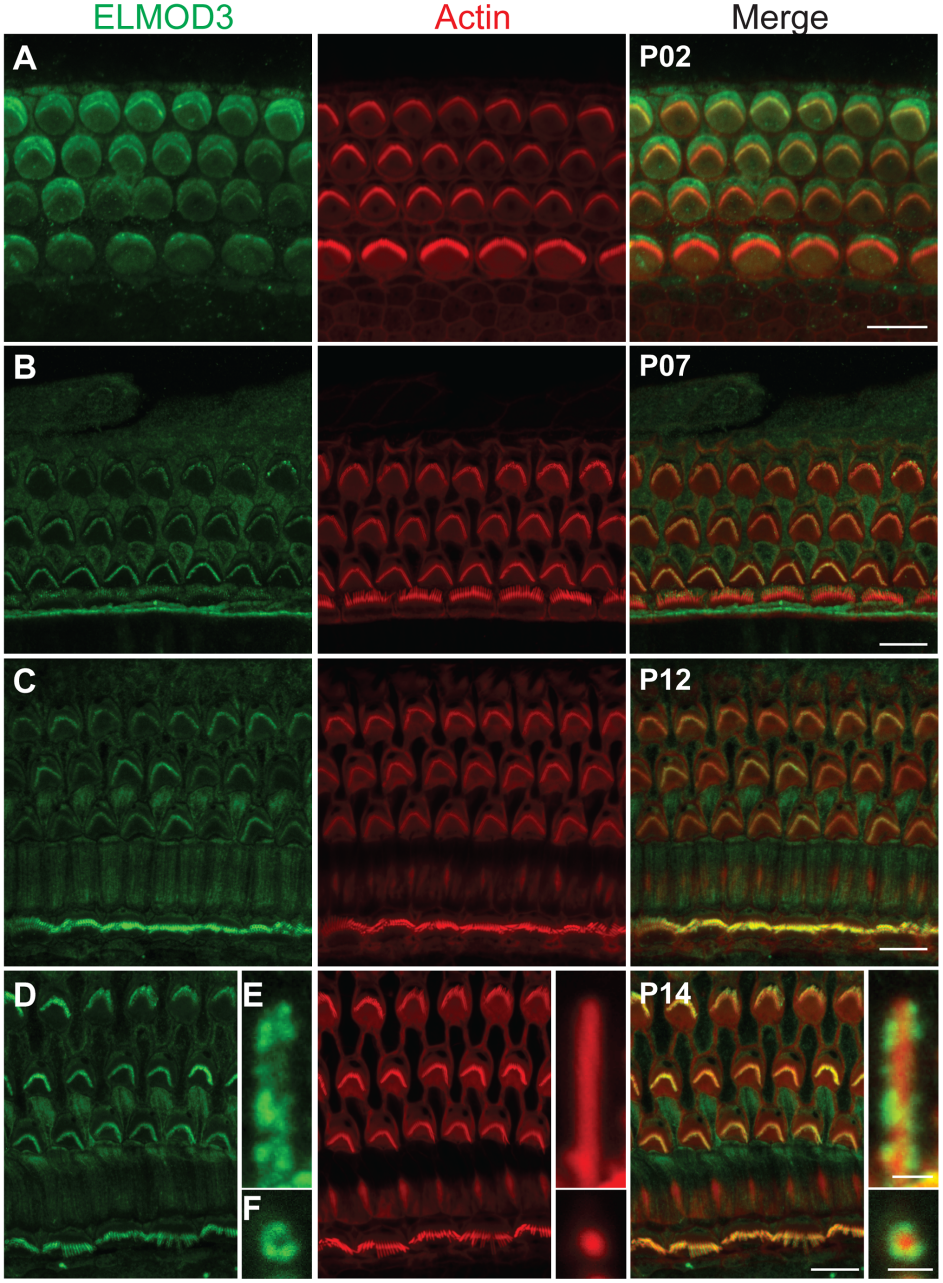
ELMOD3 is expressed in sensory cells of rat organ of Corti. (A–F) The spatio-temporal expression pattern of ELMOD3 in the rat organ of Corti. (A) At P02, ELMOD3 immunoreactivity (green) was found in the stereocilia of outer hair cells and in the kinocilia of both outer and inner hair cells. ELMOD3 immunostaining was also concentrated at the cuticular plate level of auditory hair cells. Rhodamine-phalloidin (red) staining was used to label stereocilia and actin cytoskeleton of cochlear inner hair cells and three rows of outer hair cells. (B–D) Later in development, ELMOD3 immunostaining was detected in the stereocilia bundles and microvilli of sensory cells and also in the supporting cells bodies. Longitudinal (E) and top view (F) cross-sections of stereocilia of inner hair cells, at higher magnification, showed that ELMOD3 immunostaining is distributed in patches all along the length of the stereocilia but is absent from the very tip of each stereocilium. Scale bars in panels A, B, C and D are 10 µm. Scale bar in panels E–F is 1 µm. Images in panels A to D are projections of confocal optical sections, while images in panels E and F are single plane confocal sections.

**Figure 4:**
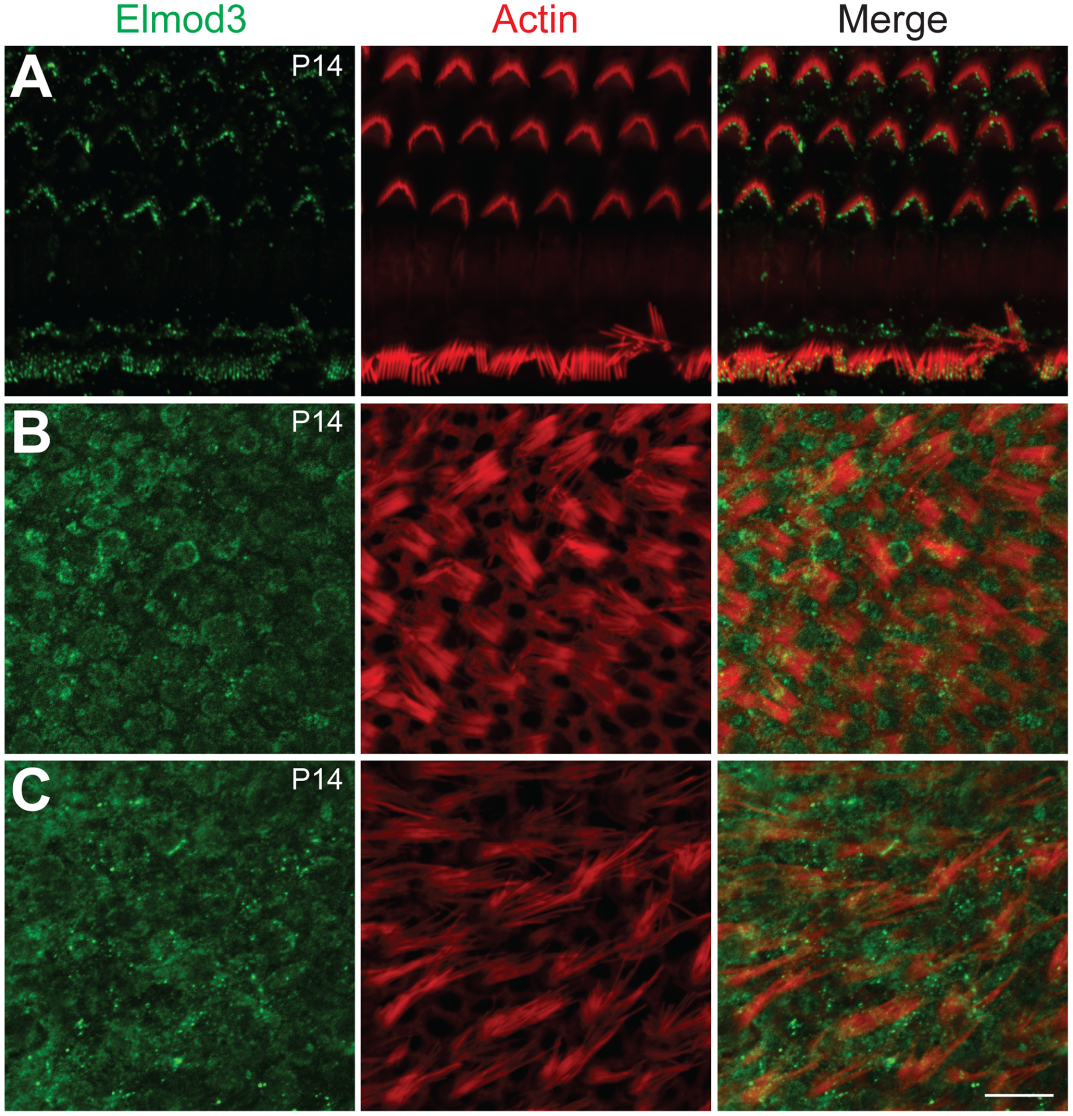
Immunolocalization of ELMOD3 in the mouse organ of Corti and vestibular sensory epithelia. (A–C) Double staining for F-actin (red) and ELMOD3 (green) in the mouse inner ear epithelia. (A) In the organ of Corti, at P14, ELMOD3 immunoreactivity (green) was detected along the length of stereocilia of hair cells, with prominent staining in the upper half of the actin-filled structures. Staining was also observed in the microvilli on the apical surface of the hair cells. (B–C) In the vestibular end organs, ELMOD3 immunostaining was localized within the hair cell and supporting cell bodies in the saccule (B) and utricule (C) at P14, but no immunoreactivity was observed in the vestibular hair bundles. All images are projections of confocal optical sections stack. Scale bar applies to all panels and is 10 µm.

To characterize the cellular localization of ELMOD3, we produced a rabbit polyclonal antiserum against synthetic peptide immunogens from mouse ELMOD3 isoform b. The sensitivity and specificity of the ELMOD3 antibody was validated in immunoblot and immunofluorescence analyses, in transfected cells and mouse tissues (Figures S4 and S7). Our antibodies specifically recognized ELMOD3 isoform b but not murine ELMOD1, ELMOD2, or ELMOD3 isoform a (Figure S4). We next performed immunolocalization of ELMOD3 in the rat and mouse organ of Corti (Figures 3 and 4). In rat cochlea, ELMOD3 immunoreactivity was observed in the stereocilia, kinocilia and cuticular plate of developing hair cells (Figures 3 and S5). Before P07, ELMOD3 staining was very weak in the inner hair cells stereocilia. By P07, in auditory hair cells, patchy labeling of ELMOD3 immunostaining was detected along the length of stereocilia (Figure 3). In contrast to actin staining, ELMOD3 immunoreactivity was not uniform along the length of each stereocilium and the protein seemed to be excluded from a region near the tip (Figure 3E). ELMOD3 immunoreactivity was also found in the supporting cells, including pillar and Dieters' cells (Figure 3). Similar to that seen in the rat (Figure 3D), the stereocilia of inner hair cells in the mouse organ of Corti were more intensely labeled than those of outer hair cells (Figure 4A). In contrast to the cochlear hair cells, ELMOD3 antibody labeling was observed within the hair cell bodies in the vestibular end organs of both rat and mouse inner ear, but no prominent immunoreactivity was observed in the hair bundles (Figures 4B–4C and S6). These observations suggest a unique role for ELMOD3 in cochlear sensory cells and may reflect the functional or structural differences between cochlear and vestibular hair bundles. No specific immunoreactivity was observed when the primary antibody was omitted (data not shown) or when the antibody was pre-incubated with the ELMOD3 peptide antigen (Figure S7).

### p.Leu265Ser mutation affects the localization of ELMOD3 in CL4 cells and mouse inner ear explants

**Figure 5:**
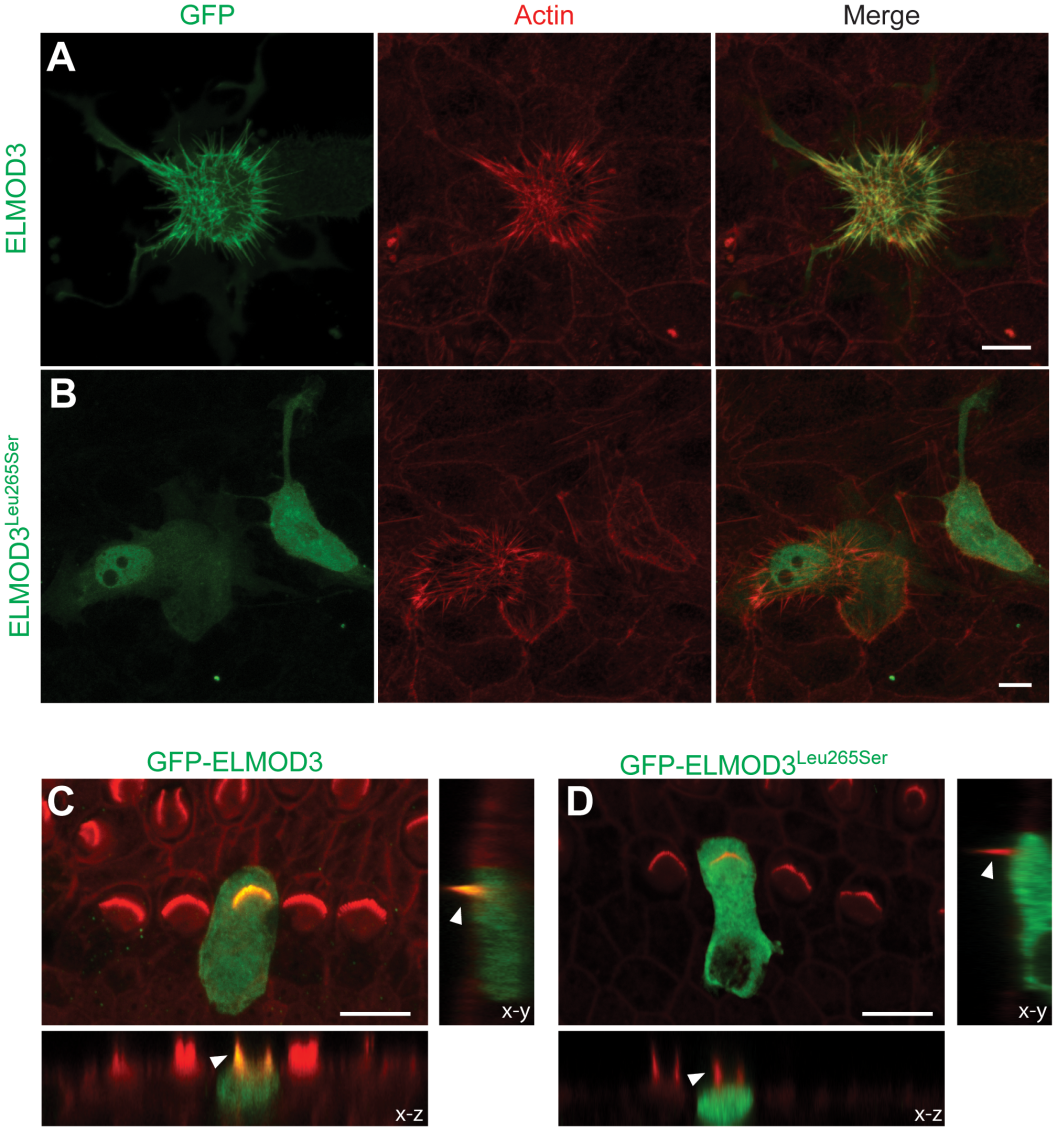
ELMOD3 accumulates at actin-based structures. (A–B) GFP-ELMOD3 localizes on actin structures (elongated microvilli due to co-expression of espin) but the deafness-causing ELMOD3 mutant does not. (A) CL4 epithelial cells were co-transfected with GFP-ELMOD3 and un-tagged Espin expression vectors. Rhodamine phalloidin (red) was used to reveal F-actin and highlight the microvilli on the CL4 cell surface. GFP-ELMOD3 was efficiently targeted to the microvillar actin bundles and the cell membrane. (B) GFP-ELMOD3 harboring the p.Leu265Ser human deafness-associated mutation retained weak microvillar targeting and cell membrane localization; the protein remained in the cytoplasm and accumulated in the nucleus. (C–D) Representative results of the transfection of P2 C57BL/6J mouse inner ear sensory epithelial explants with expression vectors that encoded either GFP-ELMOD3 or p.Leu265Ser ELMOD3. The *x–z* and *y–z* plane projections are also presented (lower panels). (C) An inner hair cell (IHC) in the mouse organ of Corti that was transfected with GFP-ELMOD3 reveals localization along the length of stereocilia (arrowhead) and throughout the hair cell body. (D) Mouse organ of Corti hair cell that was transfected with GFP-ELMOD3 (p.Leu265Ser). No concentration and only negligible fluorescence is observed in the stereocilia (arrowhead), and mutated ELMOD3 remains in the cytosol. Scale bar applies to all panels and is 10 µm.

We examined LLC-PK1-CL4 epithelial (CL4) cells to understand the mechanism and effect of the hearing loss-associated allele of *ELMOD3*. CL4 cells contain actin-rich microvilli and have been used as *in vitro* models of stereocilia to examine F-actin and protein dynamics [25]. We transiently co-transfected GFP-*ELMOD3* constructs with *Espn* constructs, where the latter was used to over-elongate the microvilli at the CL4 cell surface [26] (Figure 5A–5B). We observed a significant expression of GFP-ELMOD3 in the apical (microvillar) plasma membrane twenty-four hours post-transfection (Figure 5A). We also observed expression of GFP-ELMOD3 in the cytosol of transfected cells (Figure 5A). In contrast to the wild type protein, the p.Leu265Ser mutation in the ELMO domain yielded a protein that displayed either weak or no labeling in the microvilli of the transfected CL4 cells. Additionally, the protein appeared to be diffusely located throughout the cytoplasm, with a nuclear concentration in approximately half of the transfected cells (Figure 5B). Identical results were observed with tdTomato-tagged wild-type and mutant *ELMOD3* constructs (data not shown).

To determine the effect of p.Leu265Ser mutation on the localization of ELMOD3 in the mouse inner ear, we performed gene gun-mediated transfection of wild-type and p.Leu265Ser mutant GFP-tagged *ELMOD3* cDNA constructs in organotypic cultures of inner ear sensory epithelia of P2 C57BL/6J mice (Figure 5C–5D). Over-expressed wild-type GFP-ELMOD3 localized along the length of the stereocilia of cochlear hair cells (Figure 5C). We also observed homogeneous distribution throughout the hair cell bodies (Figure 5C). Similar to the results that were observed in CL4 cells, GFP-ELMOD3 harboring the p.Leu265Ser mutation failed to target to the stereocilia, and the protein was apparently distributed throughout the cochlear hair cell bodies (Figure 5D). Taken together, these results support our conclusion that ELMOD3 localizes to actin-based microvilli and stereocilia (Figure 5) but that a point mutation in the ELMO domain can prevent its normal localization and potentially affect its function in the stereocilia.

### ELMOD3 is linked to the actin cytoskeleton

**Figure 6:**
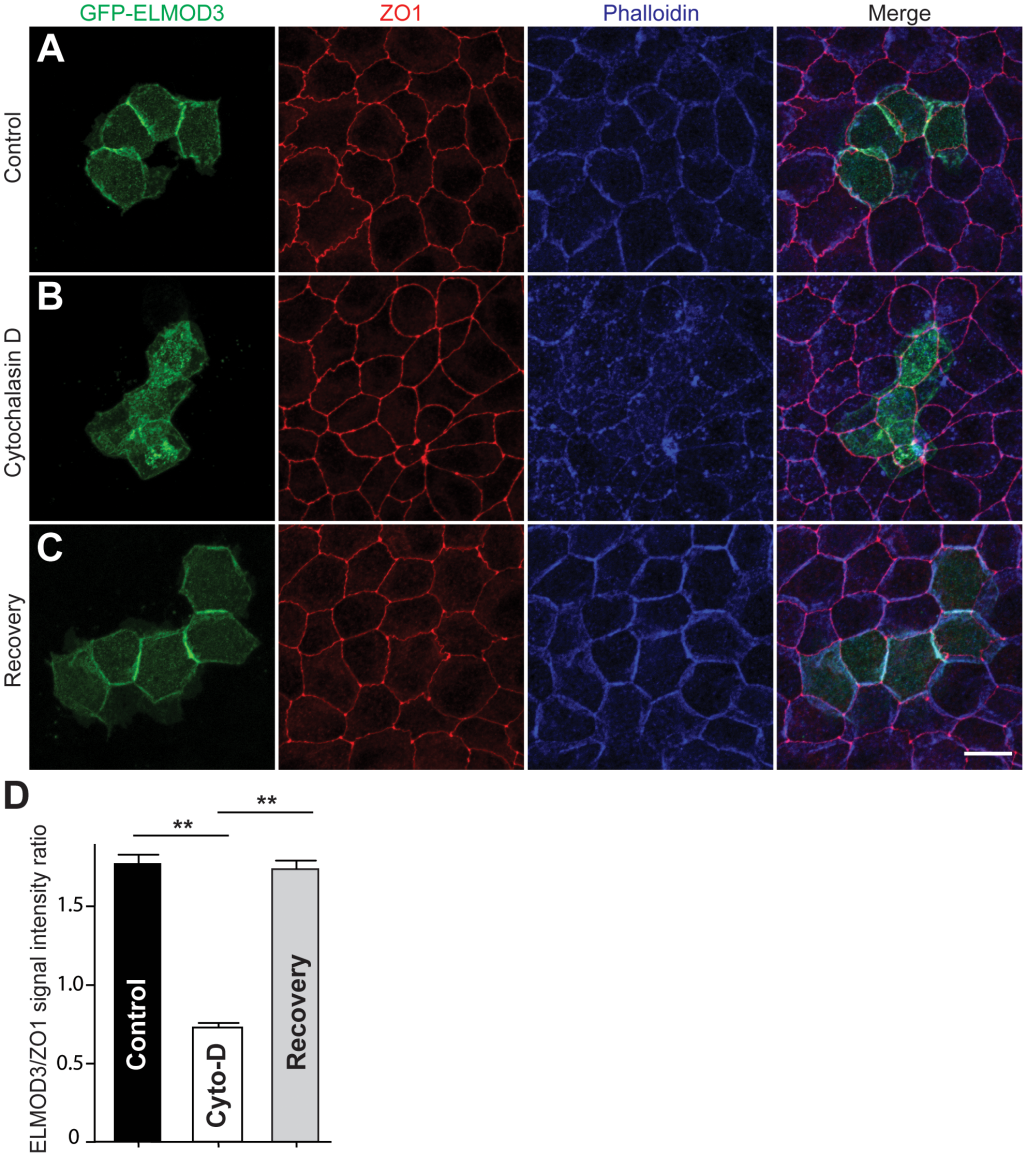
ELMOD3 is linked to the F-actin cytoskeleton. (A) In transfected MDCK cells, GFP-ELMOD3 (green) localized with F-actin at the cell membrane. Alexa647-phalloidin (blue) was used to stain F-actin, and a ZO1 antibody was used to label the tight junctions (red). (B) When the cells were treated with cytochalasin D (Cyto-D), GFP-ELMOD3 was internalized, and no or negligible localization at the cell membrane was observed. (C) Four hrs post-Cyto-D treatment, GFP-ELMOD3 re-localized with the actin cytoskeleton at the plasma membrane. Scale bar applies to all panels and is 10 µm. (D) Quantification of membranous GFP-ELMOD3 localization. The normalized ratio of GFP-ELMOD3 and ZO1 signal intensities at the cell membrane (n = 30, ** p<0.01) confirmed the internalization of ELMOD3 following Cyto-D treatment.

To further investigate the ELMOD3-actin association, we transfected GFP-tagged ELMOD3 into MDCK cells, which is a highly polarized cell model system (Figure S8). Forty-eight hours post-transfection, GFP-ELMOD3 accumulation was apparent at the periphery of the transfected cells near the plasma membrane (Figure S8A). The expression of GFP-ELMOD3 harboring the p.Leu265Ser mutation in MDCK cells resulted in a protein that failed to target or accumulate at the plasma membrane and instead, appeared to concentrate in the nuclei (Figure S8B). To determine whether GFP-ELMOD3 associates with the actin cytoskeleton at the plasma membrane (Figure 6A), we treated the cells with cytochalasin D (cyto-D), which is a potent inhibitor of actin polymerization, to disrupt the actin cytoskeleton [27], [28]. We hypothesized that if GFP-ELMOD3 associated with the actin cytoskeleton at the cell membrane, then treatment of the cells with cyto-D would also affect ELMOD3 localization. Indeed, we observed a significant decrease in the GFP-ELMOD3 signal at the cell membrane following disruption of the actin cytoskeleton (Figure 6B, 6D). Four hours following cyto-D treatment (i.e., the recovery period for actin re-polymerization) [27], we observed that ELMOD3 re-accumulated at the cell membrane (Figure 6C, 6D). These results suggest that the localization of ELMOD3 is dependent on the actin cytoskeleton and/or may contribute to a mechanism that supports its maintenance.

**Figure 7:**
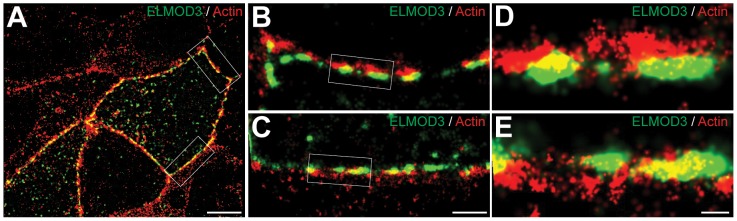
ELMOD3 is localized at the plasma membrane. STORM imaging revealed close proximity between actin filaments and over-expressed GFP-ELMOD3 at the plasma membrane in MDCK cells. (A) A representative two-color STORM image of transfected MDCK cells is shown. Alexa647 phalloidin was used to label F-actin and a Cy3B-conjugated GFP antibody was used to detect GFP-ELMOD3. In most places the GFP-ELMOD3 (green) signal did not co-localize with actin (red) at the plasma membrane. However, at few spots we found partial overlap between ELMOD3 and actin. (B–C) Magnification of the regions inside the boxes in A. (D–E) Zoomed-in views of the regions inside the boxes in B and C, respectively. Scale bars: 5 µm for A, 1 µm for B and C, 200 nm for D and E.

To decipher the link between F-actin and ELMOD3, we performed a two-color stochastic optical reconstruction microscopy (STORM) imaging of EGFP-ELMOD3 transfected MDCK cells. While conventional confocal acquisitions revealed co-localization of ELMOD3 and the actin-cytoskeleton, this high resolution imaging technique allowed us to determine more precisely the relative positions of ELMOD3 and actin (Figure 7). ELMOD3 and actin were each found in close apposition to the plasma membrane and in irregularly shaped puncta (Figure 7C). Many regions of extensive overlap in staining between ELMOD3 and actin, suggest the possibility that a subset of the actin-based structures may contain ELMOD3 but that each protein is also found localized independently of the other at the plasma membrane (Figure 7C).

To test the possibility that ELMOD3 binds to actin-based structures, we performed a high-speed co-sedimentation assay that pellets actin along with its associated proteins. To obtain a source of purified ELMOD3, His6-Trigger factor-ELMOD3 (TF-ELMOD3) fusion protein was expressed in bacteria, and the recombinant protein was purified by Ni-NTA chromatography. The TF-ELMOD3 or control proteins were incubated with polymerized F-actin and subjected to high-speed centrifugation at 150,000× *g* for 1.5 hrs (Figure S9). TF-ELMOD3 co-sedimented, albeit weakly or incompletely, with F-actin in this assay (Figure S9). Under these conditions, the p.Leu265Ser mutation did not significantly impact the level of TF-ELMOD3 that co-sedimented with F-actin (Figure S9).

### ELMOD3 exhibits GAP activity against Arl2

Human ELMOD1 and ELMOD2 each possess Arl2 GAP activity [29]. We therefore investigated whether ELMOD3 also possesses GAP activity against Arl2. Previous tests of bacterially expressed human ELMOD3 as either maltose binding proteins or trigger factor fusion proteins were negative, but the homologous preparations of ELMOD1 and ELMOD2 were found to possess very low specific activities as Arl2 GAPs, compared to the preparation purified from bovine tissues. To obtain a potentially more active preparation of human ELMOD3, we expressed ELMOD3 and the mutant p.Leu265Ser in HEK293T cells as N-terminal GST-fusion proteins to facilitate protein purification. Protein expression and purification from ∼10^8^ HEK293T cells that expressed GST-ELMOD3 or the mutant each yielded ∼0.6 mg protein. These preparations were stable at 4°C and against freeze-thaw cycles, as judged by either GAP activity or lack of precipitation. We expressed and purified GST alone and used it as a negative control in all of our assays. We also evaluated the effect of cleavage of the GST fusion tag by TEV protease on ELMOD3 activity; no changes in activity in the Arl2 GAP assay were observed compared to un-cleaved proteins (data not shown). Thus, we believe that the presence of the GST moiety at the N-terminus does not interfere with access to the substrate or with enzymatic activity in our assay.

**Figure 8:**
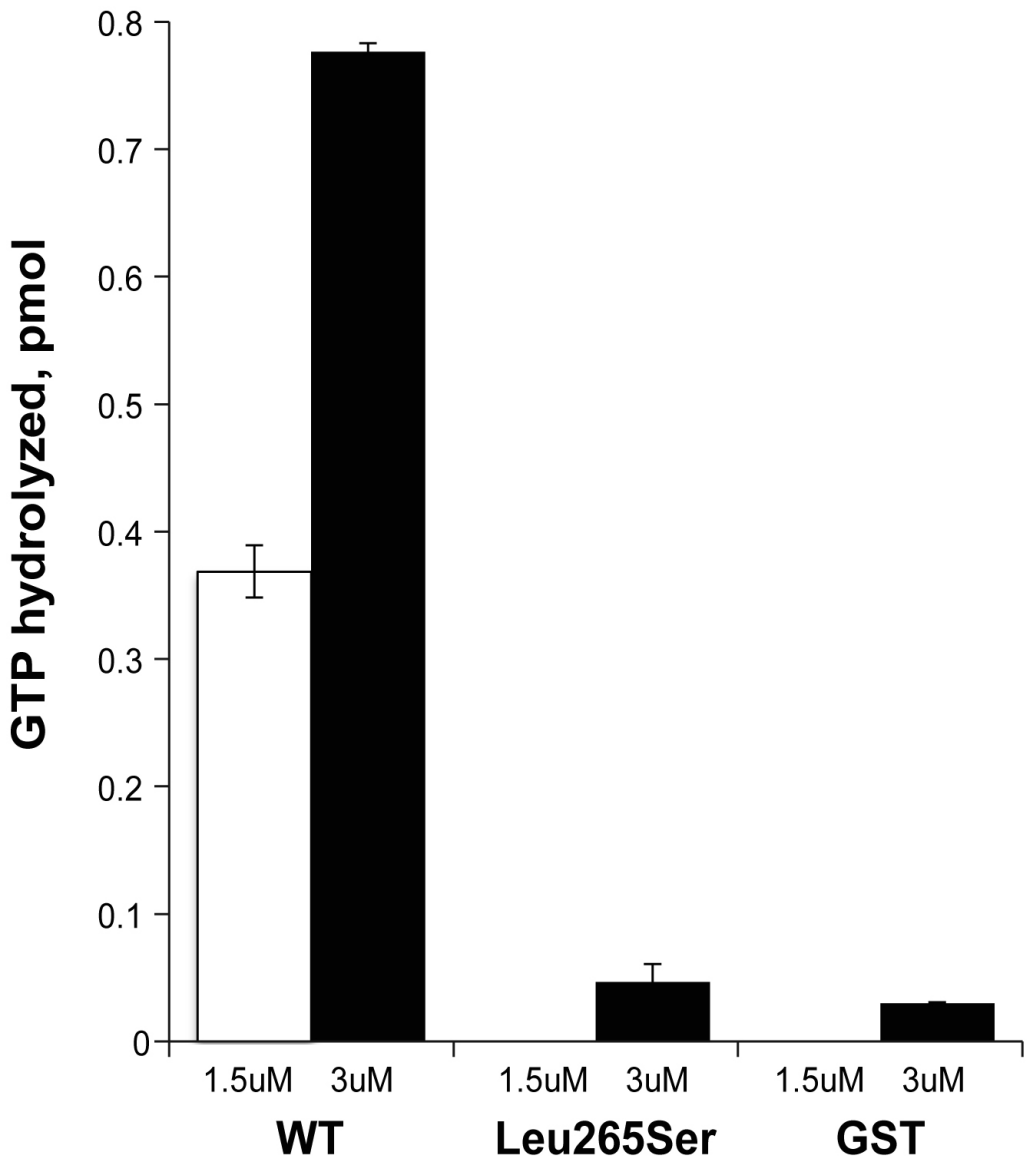
ELMOD3 has Arl2 GAP activity. Human GST-ELMOD3 (WT), the point mutant (Leu265Ser), and GST alone (GST) were expressed and purified from HEK293T cells. The white and black bars represent the Arl2 GAP activities, with 1.5 µM or 3 µM of purified protein, respectively, assayed using 200 nM Arl2 as substrate. The activities shown are the averages of duplicate experiments. The bars indicate the range. This experiment was repeated with a different preparation, and essentially identical results were obtained.

When we varied the amount of GST-ELMOD3 protein in the Arl2 GAP assay, we observed a dose-dependent response and evidence of saturation at higher protein concentrations (data not shown). Using the lower concentrations of GST-ELMOD3 to estimate the initial rates of GAP-dependent activity and estimating the purity of the preparation at 50% (based on visual inspection of Coomassie blue-stained gels), we obtained a specific activity of 24 pmol of GTP hydrolyzed/min/mg (Figure 8). This specific activity of GST-ELMOD3 as an Arl2 GAP is approximately 32-fold lower than that determined for GST-ELMOD1 and nearly 1000-fold lower than that of GST-ELMOD2 or bovine testes ELMOD2, which is the most active reported preparation of any Arl2 GAP [29]. Thus, in contrast to our earlier report that it is inactive, GST-ELMOD3 does exhibit Arl2 GAP activity, and we believe that its lower specific activity when expressed in bacteria likely contributed to the earlier negative findings [29]. The differences in specific activity among the three human GST-ELMOD preparations from HEK293T cells are predicted to result from differences in substrate specificity, sensitivities to co-activators (as known for Arf GAPs), or both. Thus, more studies are required to determine whether the biologically relevant substrate of ELMOD3's GAP activity in the inner ear is Arl2 or a related GTPase.

We next assessed the effects of the Leu265Ser point mutation on the Arl2 GAP activity of GST-ELMOD3. The mutant protein was expressed at the same levels in HEK293T cells and was purified in the same way, resulting in equivalent amounts of protein, indicating that the protein is equally stable in mammalian cells and in solution. However, when assayed for Arl2 GAP activity, the mutant was inactive (Figure 8). Although we observed small amounts of activity over our no protein control, this level of activity seen for the mutant was not different from that observed with GST alone (Figure 8). These activities are so low as to be at or near the lower limits of our assay. Thus, we can safely conclude that the point mutant has at least a 10-fold lower specific activity than the wild-type protein, but it might be completely inactive as an Arl2 GAP.

## Discussion

Our study revealed that *ELMOD3* is important for hearing in humans as a missense mutation in the gene leads to profound hearing impairment. ELMOD3 belongs to the engulfment and cell motility (ELMO) protein family, which includes six known members in mammals (ELMO1-3 and ELMOD1-3). Our *ex vivo* studies reveal that fluorescently tagged ELMOD3 localized with the actin-based microvilli of LLC-PK1-CL4 epithelial cells, in the stereocilia of sensory hair cells of mouse organ of Corti explants, and to a lesser extent to the actin cytoskeleton of MDCK cells, whereas the deafness-associated allele (p.Leu265Ser) was deficient in each case. Similarly, we show that human ELMOD3 possesses Arl2 GAP activity but the mutant has at least a 10-fold loss in activity.

While ELMOD3 antibody reactivity was detected in outer hair cells stereocilia at P02, more pronounced accumulation of ELMOD3 immunoreactivity was detected in rat cochlear inner hair cell stereocilia only by P12, which is when hair bundles are in the late phase of maturation. During this period, the inner hair cell stereocilia undergo a rapid elongation [30]. The observed staining suggests that ELMOD3 might be necessary for the initial development of the outer hair cell stereocilia or the organization of the bundle in a staircase pattern but may play a different role in the stereocilia of inner hair cells. Nevertheless, it is tempting to speculate that ELMOD3 may play a role in the maturation or maintenance of the cochlear stereociliary bundle. Recently, two spontaneous mutations (*rda* and *rda^2J^*) in mouse *Elmod1* were shown to result in profound deafness and vestibular dysfunction [7], demonstrating that the function of ELMOD1 is essential for regulating the shape and maintenance of inner ear hair cell stereocilia in mice [7]. *Elmod1* has been shown to be part of a large cluster of genes expressed in the developing inner ear, while *Elmod3* level was below the detectable range [31]. These observations are consistent with findings from other studies, like the SHIELD database, which demonstrated that the level of *Elmod3* mRNA is ∼100-fold lower than that of *Elmod1* in the developing inner ear (P0–P1).

Besides stereocilia bundles, immunoreactivity for ELMOD3 was also detected along the kinocilium in developing cochlear hair cells. Recently, it has been shown that ELMO1 can act at the interface between the actin-cytoskeleton and microtubule network by interacting with ACF7 (Actin crosslinking family 7) [32]. Moreover, microtubule polymerization depends on Arl2 activity [33], and we have shown that ELMOD3 exhibits a GAP activity against Arl2. Therefore, ELMOD3 expression in the kinocilium might have a role in the assembly of the kinocilium architecture and in pathways regulating planar cell polarity.

The ELMO family proteins are functionally poorly characterized, and more information is currently available for the ELMOs than for the ELMODs, with no structural information available for any of them. So far, only one activity has been ascribed to ELMOD proteins: we previously reported that recombinant human ELMOD1 and ELMOD2 display *in vitro* Arl2 GAP activity, whereas ELMOD3 and bacterially expressed ELMO1-3 did not [29]. More recently, we performed additional phylogenetic and functional analyses of the ELMO domain that led us to re-examine whether ELMOD3 shares the Arl2 GAP activity of ELMOD1 and ELMOD2. Our data contrast with the earlier-published claim that ELMOD3 lacks Arl2 GAP activity; we determined that it does indeed possess this activity, albeit at a substantially lower specific activity than that of its two closest human paralogs. The large differences in specific activities observed in the Arl2 GAP assay may be due to differences in the specificities of ELMODs as GAPs for different GTPases, including the lack of one or more binding partners (e.g., one that is perhaps analogous to Dock180 binding to ELMO1 or a co-activator for activity such as has been proposed for COP-I and ArfGAP1 [34]), and/or the lack of post-translational modification.

Our *in vitro* experiment revealed that ELMOD3 harboring the p.Leu265Ser mutation, unlike ELMOD3, has no or few GAP activity against Arl2. The lack of Arl2 GAP activity of the mutant may suggest a reduced affinity for the Arl2 GTPase, which may play an important role in ELMOD3 localization. *Elmod3* and *Arl2* are expressed in developing mouse cochlear tissues and weakly in vestibular tissues (Figure S10). Even though ELMOD3 is active and has been defined as “Arl2 GAPs”, we expect it to be active against other GTPases in the Arf family as well. We therefore speculate that ELMOD3 functions as a GAP for Arl2 and perhaps other GTPases that participate in actin organization, polymerization or depolymerization in the cochlear hair bundles. If ELMOD3 is an active GAP for other GTPases, these GTPases are likely to be part of the Arf family given that GAPs are not known to cross family boundaries within the Ras superfamily. However, it is plausible that ELMOD3 functions in a signaling pathway that includes Arl2 (or an Arf family GTPase), Rac, Rho, and, ultimately, affects the actin cytoskeleton.

Future studies will address the specific roles of ELMOD3 in the development of the inner ear sensory epithelium, cytoskeletal organization, and ELMOD3-mediated signaling pathways. Revealing the interacting partners, substrate specificities for its GAP activities, as well as the means of regulation of ELMOD3 and other ELMO family proteins, will shed light on the overlapping functions of the Ras superfamily in the inner ear. These fundamental functions of this unique protein family are likely to be important in all eukaryotic cells.

## Materials and Methods

### Family ascertainment and phenotype analysis

Family PKDF468 was enrolled in the present study from the Punjab province of Pakistan, and written informed consent was obtained from all participating family members. The Institutional Review Boards at the Center for Excellence in Molecular Biology (Pakistan), at the National Institute on Deafness and Other Communication Disorders, and at Cincinnati Children's Hospital (USA) approved the present study. Hearing loss in the affected family members was evaluated using pure-tone audiometry, which tested frequencies that ranged from 125 Hz to 8 kHz. The family medical history stated that the onset of hearing loss was pre-lingual, and we observed no evidence of vestibular dysfunction or other balance issues using the Romberg and tandem gait tests. There were no other significant findings from the clinical exam, and the affected members had basic metabolic panel results within the normal range, indicating that they had nonsyndromic hearing loss.

### Linkage analysis and whole exome sequencing

We conducted a genome-wide scan on family PKDF468 using 388 STR markers and performed linkage analysis using GeneMapper software (Applied Biosystems; Carlsbad, CA). The LOD score was calculated using a recessive model of inheritance assuming a fully penetrant disorder and a disease allele frequency of 0.001. The primers were designed with Primer3 to sequence all of the coding exons and 75 bp of the exon-intron boundaries of all of the known genes within the *DFNB88* locus (Table S2). The products were amplified using either Taq polymerase (Genscript; Piscataway, NJ) or Amplitaq Gold 360 (Applied Biosystems) for the GC-rich regions. The chromatograms were read using SeqMan software (DNAStar; Madison, WI).

Exome sequencing was conducted on one affected individual from family PKDF468 and was enriched using the Nimblegen SeqCap EZ Exome v2.0 Library (Roche Diagnostics; San Francisco, CA). One hundred base pair paired-end sequencing was performed on an Illumina Hi-Seq 2000 system. The sequencing data were analyzed following the guidelines that are outlined in the Broad Institute's Genome Analysis Toolkit [35], [36]. The row data were mapped using the Burrows Wheeler Aligner [36], the variants were called using the Unified Genotyper, and the data underwent further processing and quality control [35], [36]. Low-quality reads (less than 10× coverage) were removed, and the remaining variants were filtered against the dbSNP133 database and all of the known variants in the NHLBI 6500 Exome Variant database that had a minor allele frequency (MAF) of greater than 0.05%. We also filtered out additional variants that were observed in six ethnically matched control exomes. Primers were designed, using Primer3, to screen the remaining candidate gene variants, and we performed segregation analysis by performing Sanger sequencing of the variants of all of the participating family members.

### *ELMOD3/Elmod3* expression analyses

Human and mouse *ELMOD3/Elmod3* isoform-specific primers and TaqMan probes were designed, using Primer3 web-based program, and the transcripts were amplified from human and mouse cDNA libraries (Clontech Laboratories; Mountain View, CA).

Mouse inner ear tissues were harvested from 3 or more C57BL/6J mice at P0, P10, and P30. The cochlea and vestibular system were separated from the inner ear, and the total RNA was extracted from each tissue using TRIreagent (Life Technologies, Grand Island, NY). The RNA was reverse-transcribed into cDNA using the SMARTscribe Kit (Clontech). Real-time PCR was performed in triplicate on a StepOne Plus instrument (Applied Biosystems). The data were analyzed using the comparative Ct method, with *Gapdh* as the endogenously expressed reference gene. RT-PCR was performed by using LA Taq (Clontech). The products were run on a 2% agarose gel that was stained with ethidium bromide and each isoform was verified by sequencing.

To determine the *in vivo* effect of c.794T>C allele, if any, on the splicing of *ELMOD3* transcripts, total RNA was isolated from fresh blood samples of two affected individuals (V:2 and V:11) and one normal hearing individual (V:3) by use of TRIzol reagent (Life Technologies). Oligo dT and randomly primed first strand cDNA libraries were generated using SMART 1^st^ strand cDNA synthesis kit (Clontech). Touchdown PCR was performed with GenScript Taq (GenScript) and 1.5 mM MgCl_2_ at an annealing temperature of 63°C for 30 cycles using *ELMOD3*-specific primer pairs with a common forward primer in exon 9 and reverse primer either in exons 11 (*hELMOD3_ex9-11*; Table S5) or in exon 12 (*hELMOD3_ex9-12*; Table S5). *GAPDH* was used as a control and amplified under the same conditions. PCR fragments were subcloned into pCR-TOPO cloning vector (Life Technologies), and the sequences were verified.

### *ELMOD3* expression constructs

Human *ELMOD3 B* isoform, murine *Elmod1*, *Elmod2* and *Elmod3* isoforms *a* and *b* open reading frame have been amplified from commercially available human and mouse cDNA libraries (Clontech) and inserted in pEGFP-C2 vector (Clontech) to generate proteins with GFP fused to their N-termini. The construct encoding p.Leu265Ser ELMOD3 was prepared through site-directed mutagenesis (Agilent Technologies, Santa Clara, CA) using the wild-type ELMOD3 isoform B as a template. The full-length open reading frame of human ELMOD3 (isoform B) was cloned into the pCOLD-TF (Takara Bio, Inc.; Otsu Shiga, Japan) vector at the BamHI and SalI sites by PCR amplification of the cDNA using primers that inserted the appropriate restriction sites. This generated a fusion protein with a His6 tag at the N-terminus. This tag was followed by trigger factor (∼48 kDa), a thrombin cleavage site, and the ELMOD3 open reading frame. To insert the Leu265Ser mutation, we performed site-directed mutagenesis on the construct using the QuikChange Lightening Kit (Agilent Technologies). Full-length open reading frames of human ELMOD3 (Isoform B) and the Leu265Ser mutant were cloned into the pLEXm-GST vector [37] using KpnI and SphI sites that were inserted into the PCR primers, with subsequent confirmation of the correct DNA sequence. The parent vector was used to express GST alone.

### Inner ear explant and gene gun

Inner ear explants were harvested from C57BL/6J mice at P2. The explants were cultured in a glass-bottom Petri dish (MatTek, Ashland, MA) that was coated with Matrigel (BD Biosciences, San Jose, CA) and were maintained in DMEM that was supplemented with 7% fetal bovine serum (FBS) (Life Technologies) for 24 hrs at 37°C with 5% CO_2_. The cultures were transfected using a Helios gene gun (Bio-Rad, Hercules, CA), as described elsewhere [38].

### Cell culture and transfection

HEK293T, CL4 and MDCK cells were grown in DMEM that was supplemented with 10% FBS, 2 mM L-glutamine, and penicillin/streptomycin (50 U/ml) (Life Technologies) and were maintained at 37°C in 5% CO_2_. The cells were transfected using Fugene HD Transfection Reagent (Promega; Sunnyvale, CA), according to the manufacturer's instructions. The cells were then cultured for an additional 48 hrs prior to immunostaining. Forty-eight hrs following transfection, we added 2.5 µM Cytochalasin D (EMD Millipore, Billerica, MA) in fresh DMSO medium to the cells for two hrs to disrupt the actin cytoskeleton. The Cytochalasin D was then washed out, and the cells were grown for four additional hrs in complete medium.

### Antibody

ELMOD3 antiserum was raised in rabbits against two synthetic mouse ELMOD3-specific peptides (corresponding to residues 143–156 and 346–361 of the mouse sequence [GenBank accession number GI:358679299]). The immunizations and sera collections were performed by Covance (Princeton, New Jersey). The antiserum was affinity purified (AminoLink Plus Immobilization Kit; Thermo Scientific, Rockford, IL) either using both synthetic peptides in combination or individually.

### Western blot

Antibody specificity was assessed by transfections (Fugene HD Transfection Reagent; Promega) of GFP-tagged mouse ELMOD1, ELMOD2 and ELMOD3 into HEK293T cells followed by western blot analysis, as described elsewhere [39]. Inner ear and olfactory bulbs tissues were harvested from C57BL/6J mice at P30 and followed by western blot analysis, as described elsewhere [39]. Antigen competition was performed by incubating the primary antibody for 30 min at room temperature with the two immunizing peptides prior to use in Western Blot or immunofluorescence analyses.

### Immunostaining

C57BL/6J mice were obtained from Jackson Laboratories (Bar Harbor, ME) and bred in house. Sprague–Dawley rats were purchased from Charles River Breeding Laboratories (Raleigh, North Carolina) and bred in house. All experiments and procedures were approved by the Institutional Animal Care and Use Committee of the Cincinnati Children's Hospital Medical Center.

The inner ears from rats and mice were fixed with 4% PFA at 4°C overnight. P12 and P14 rat cochlea were incubated for one day, at 4°C, in 0.25M EDTA. The sensory epithelia were dissected in PBS. Following permeabilization with 0.25% Triton X-100 for 45 min, the samples were incubated in blocking solution (5% normal goat serum in PBS). The samples were then incubated overnight at 4°C with primary antibody (anti-ELMOD3; anti acetylated Tubulin (Sigma-Aldrich, St Louis, MO)) in 3% NGS/PBS. This step was followed by three washes in PBS and consecutive incubation with Alexa-488 conjugated secondary antibody and Alexa-647 conjugated secondary antibody (Life Technologies) at 1∶500 dilution and with rhodamine phalloidin (Life Technologies) at 1∶200 dilution in 3% NGS/PBS for one hour. After three washes with PBS, the samples were mounted using ProLong Gold Antifade Reagent (Life Technologies).

Transfected cells and inner ear explants were washed with PBS and fixed for 20 min in 4% paraformaldehyde. Filamentous actin was detected with rhodamine phalloidin (Life Technologies) in PBS/0.1%Triton-X100 for one hour. Following subsequent washes, the coverslips were mounted using FluoroGel medium (Electron Microscopy Sciences, Hatfield, PA) for the cell monolayers or with ProLong Gold Antifade Reagent (Life Technologies) for the explant cultures.

MDCK cells were washed with PBS, fixed for 20 min in 4% paraformaldehyde and blocked with 10%NGS/PBS/0.1% Triton-X100. The cells were incubated overnight at 4°C with a primary antibody (ZO1, Life Technologies; anti-ELMOD3) in 3% NGS/PBS/0.1%Triton-X100. The cells were then washed in PBS/0.1%Triton-X100 and incubated with the Alexa-fluor 546 conjugated secondary antibody (Life Technologies) in 3% NGS/PBS/0.1% Triton-X100 for one hour at room temperature. Filamentous actin was detected with Alexa647-phalloidin (Life Technologies) in PBS/0.1%Triton-X100 for one hr. Following subsequent washes, the coverslips were placed using FluoroGel mounting medium (Electron Microscopy Sciences).

All images were acquired using a Zeiss LSM 700 scanning confocal microscope that was equipped with 63× and 100× objectives, and the analyses were performed using ImageJ software. The pixel intensity analyses were performed using ImageJ software on images that were acquired with the same microscope settings. The statistical analyses were performed using GraphPad Prism software and the ANOVA test function.

### Immunofluorescence and STORM imaging

Transfected MDCK cells were washed with PBS and fixed for 15 min in 4% paraformaldehyde. Following a one hour incubation in blocking solution (10% normal goat serum in PBS with 0.1% Triton X-100), the cells were then incubated one hour with primary antibody: polyclonal chicken anti-GFP (Aves Labs Inc., Tigard, OR) custom conjugated with APEX Alexa Fluor 568 Antibody (Life Technelogies), in PBS/0.1%Triton-X100/3%NGS. Filamentous actin was labeled concomitantly with Alexa647-phalloidin (Life Technologies). After extensive washes in PBS/0.1%TritonX-100/3%NGS, the cells were fixed 15 min in 4% paraformaldehyde and stored at 4°C in PBS.

For imaging, PBS was replaced by the following imaging medium: 2-mercaptoethanol, buffer B (10% glucose, 50 mM Tris-HCl pH = 8, 10 mM NaCl) and the GLOX system (14 mg Glucose Oxidase, 50 ul Catalase, 200 ul Tris buffer) in a 1∶100∶1 volume ratio. N-STORM imaging was performed with a Nikon N-STORM super-resolution microscope system (Nikon Instruments Ltd, Melville, NY) based on an inverted microscope Nikon A1Rsi equipped with a perfect-focusing system and a 100× TIRF APO NA 1.49 oil objective. A 561 nm wavelength laser was applied for bleaching and excitation of Alexa Fluor 568 while a 647 nm wavelength laser was applied for Alexa Fluor 647. The images were acquired with an Andor Xion 897 EMCCD camera using 16 ms exposition with one frame of imaging for 5000 cycles. The analysis was performed with Nikon Elements Storm software.

### Actin co-sedimentation assay

BL21 (DE3) Gold *E. coli* were transfected with the pCOLD-TF-ELMOD3 and pCOLD-TF-ELMOD3 Leu265Ser plasmids, and single colonies were used to inoculate cultures in LB medium with 100 µg/ml ampicillin, which were grown at 37°C with shaking. When OD_600_ = 0.5, the cells were moved to 15°C for 30 min without shaking. IPTG was then added to 1 mM, and the culture was grown overnight at 15°C with shaking. The cells were collected and lysed by passage through a French press in 20 mL of 20 mM HEPES (pH 7.5), 150 mM NaCl, and 5 mM imidazole with a protease inhibitor cocktail (Sigma-Aldrich). The lysates were clarified by centrifugation at 100,000× *g* for one hour at 4°C, and the supernatants were loaded onto a Ni-NTA column (GE Healthcare) that was pre-equilibrated in the same buffer. The column was washed with 20 mL of 20 mM HEPES (pH 7.5), 150 mM NaCl, and 55 mM imidazole prior to elution in 15 mL buffer that contained 250 mM imidazole. The protein was further purified and buffer-exchanged by gel filtration chromatography using a Superdex S200 column (24 mL) that was run in 20 mM HEPES (pH 7.5), 150 mM NaCl, and 1 mM dithiothreitol. Typical yields from this protocol were ∼10 mg/L TF-ELMOD3 that was >90% pure, as estimated by visual inspection of a Coomassie blue-stained gel. The actin binding experiment was performed with purified TF-ELMOD3 and TF-ELMOD3 Leu265Ser using an Actin Binding Protein Biochem Kit (Cytoskeleton Inc, Denver, CO), following the manufacturer's protocol and later repeated with the GST-ELMOD3 proteins purified from HEK cells.

### Purification of GST-ELMOD3 from HEK293T cells

GST-ELMOD3, the point mutant, or GST alone was expressed in HEK293T cells using the pLEXm-GST vector (a kind gift from Dr. James Hurley (NIDDK)) and a modification of the method that was described in Aricescu et al [37]. Briefly, HEK293T cells (10×10 cm plates) were transfected at 90% confluency with 1 µg/mL DNA after mixing with polyethyleneimine (PEI-MAX; Polysciences, Inc.; Warrington, PA) at a 1∶3 ratio of DNA∶PEI in Opti-MEM medium (Life Technologies). The mixture was then added to cells in DMEM medium that contained 2% FBS and grown for two days. The cells were collected by centrifugation and lysed by resuspension in 1.5 ml 25 mM HEPES (pH 7.4), 100 mM NaCl, and 1% CHAPS. The solution was clarified by centrifugation for 30 min in a microfuge at a maximum speed (∼14,000× g) at 4°C. Glutathione Sepharose 4B (GE Healthcare) beads were added and incubated at 4°C for 3 hrs with mixing. The beads were then pelleted, washed twice in 25 mM HEPES (pH 7.4) and 100 mM NaCl and eluted (2×0.5 mL) in the same buffer containing 20 mM glutathione. The eluted protein was concentrated to 0.25 mL in a spin concentrator (Amicon Ultra-4; EMD Millipore). The protein concentration was determined using a Bradford assay. Typical yields of preparations from 10×10 cm plates were ∼600 µg of GST-ELMOD3 or mutant and ∼6 mg of GST. The purified proteins were quick frozen and stored at −80°C.

### Arl2 GAP assays

The Arl2 GAP assay was performed as described previously by Bowzard *et al* [29]. Briefly, 2 µM purified recombinant Arl2, prepared as described by Clark *et al* [40], was pre-loaded with [γ-^32^P]GTP in 25 mM HEPES (pH 7.4), 2.5 mM MgCl_2_, 100 mM NaCl, 1 mM EDTA, 25 mM KCl, and 0.5 mM ATP in a total volume of 100 µL. The incubation was performed at 30°C for 30 min. The GAP reaction was performed in a buffer that contained 25 mM HEPES (pH 7.4), 2.5 mM MgCl_2_, 100 mM NaCl, 1 mM dithiothreitol, 2 mM ATP, 1 mM GTP and the pre-loaded Arl2 [γ-^32^P]GTP. The total 50 µL reaction was initiated by the addition of the sample that contained the Arl2 GAP and stopped after 4 min at 30°C by the addition of 750 µL of ice-cold activated charcoal (5% activated charcoal (Sigma-Aldrich, St. Louis, MO) in 50 mM Na_2_HPO_4_ (pH 7.4). The samples were clarified by centrifugation, and 400 µL was taken for counting in a liquid scintillation counter. Each GAP sample was also assayed in parallel in a tube that contained all of the above reagents except Arl2 (i.e., the same amount of [γ-^32^P]GTP). The resulting “blank” values were subtracted from the results that were obtained in the presence of Arl2 to determine the amount of hydrolyzed ^32^P_i_ that was dependent on Arl2 GAP activity. More detail and descriptions regarding how the specific activities were calculated from this assay can be found in the report by Bowzard et al [29].

## Supporting Information

Figure S1 A missense mutation in the ELMO/CED12 domain of *ELMOD3* is present in all the affected individuals of the PKDF468 family. (A) The nucleotide sequence chromatograms of exon 10 of *ELMOD3*, indicating the wild-type sequence, heterozygosity and homozygosity of the c.794T>C mutation. Nucleotide cDNA positions are given according to human *ELMOD3* isoform A (accession no. NM_032213.4). (B) The predicted protein products of the different *ELMOD3* splice variants. Both the *A* and *B ELMOD3* isoforms contain an ELMO/CED12 domain and differ only at the carboxy termini. Mouse *Elmod3* isoforms *a* and *b* are 79% and 82% identical to human *ELMOD3* isoforms *A* and *B*, respectively.
(TIF)

Figure S2 The c.794T>C mutation does not affect the splicing of *ELMOD3* isoforms. Amplified *ELMOD3* short transcripts from the cDNA libraries generated using the blood samples of affected and normal hearing individuals. *GAPDH* has been used as a positive control.
(TIF)

Figure S3 RT-PCR has been performed with human and mouse cDNA libraries. Human and mouse *ELMOD3/Elmod3* isoforms *A/a* and *B–D/b–c* are expressed in many tissues.
(TIF)

Figure S4 Our custom-made antibody against mouse recognizes mouse ELMOD3 isoform b. (A) To validate our custom-made antibody against mouse ELMOD3 in immunofluorescence, we performed a co-localization using full-length EGFP-tagged murine ELMOD3 isoform b transfected in MDCK cells. EGFP-ELMOD3 (green) staining and ELMOD3 antibody (red) staining colocalize (merge). Nuclei of transfected and non transfected cells have been counterstained with DAPI. The top panels represent a single confocal microscopy section of the cells at the nuclei level and the bottom panels a projection of all the confocal microscopy sections from the base to the apex of the cells. Scale bar: 10 µm. (B) Full-length EGFP-tagged murine *Elmod1*, *Elmod2* and *Elmod3* isoforms *a* and *b* cDNA constructs were expressed in HEK cells and Western blot analysis was performed on whole cell lysates. The GFP antibody was used as a loading and transfection control. The ELMOD3 antibody specifically recognized only the ELMOD3 isoform b and not the other known members of the ELMOD family. (C) Our custom-made antibody against mouse ELMOD3 recognizes endogenous protein from mouse tissues extracts (inner ear, olfactory bulbs). GAPDH has been used as a loading control.
(TIF)

Figure S5 ELMOD3 immunoreactivity is localized in the kinocilia of developing auditory hair cells. (A) Maximal projection of confocal optical sections stacks of rat organ of Corti, at P02. ELMOD3 was immunostained in green while kinocilia were highlighted with acetylated-tubulin antibody (red). Actin cytoskeleton was labelled with rhodamine phalloidin (gray). (B) Single plane confocal acquisition of outer hair cells at the kinocilia level. (C) Single plane confocal acquisition of inner hair cells at the kinocilia level. ELMOD3 (green) immunoreactivity was found in the kinocilia (red) of both OHC and IHC in the organ of Corti of rat, at P2. Scale bar: 10 µm. Scale bars are 5 µm for higher magnification panels of IHC and OHC.
(TIF)

Figure S6 ELMOD3 immunoreactivity (green) was found within the hair cell and supporting cell bodies in the ampulla of rat, at P14, but no immunoreactivity was observed in the vestibular hair bundles. Actin cytoskeleton was highlighted with rhodamine phalloidin (red). Scale bar: 10 µm. All images are projection of confocal optical sections stack.
(TIF)

Figure S7 To validate our custom-made antibody against murine ELMOD3 in mouse tissues, we have blocked the antibody with the immunizing peptides followed by immunostaining on mouse inner ear epithelia at P14. Under these conditions, no staining has been observed in utricule (A), saccule (B) or the organ of Corti (C) epithelia. Scale bar: 10 µm. All images are projections of confocal optical sections stacks.
(TIF)

Figure S8 ELMOD3 targeting and localization with F-actin is affected by the *DFNB88* allele. (A–B) The p.Leu265Ser mutation affected the targeting and localization of ELMOD3 to the actin cytoskeleton, stained with rhodamine phalloidin (red), at the cell membrane of MDCK cells. (C) pEGFP-C2 empty vector, which was used as a control. All the panels are confocal microscopy sections at the nuclei level. Scale bar: 10 µm.
(TIF)

Figure S9 (A) Positive and negative controls for the F-actin co-sedimentation assay. The first two lanes contain the supernatant (S) and pellet (P) fractions of F-actin alone, respectively, which were obtained using high-speed centrifugation. F-actin was primarily recovered in the pellet fraction (lane 2). As a positive control, we used α-actinin, which is known to bind to F-actin. Following ultracentrifugation, α-actinin was recovered in the supernatant fraction (lane 3). When the same amount of α-actinin was incubated with F-actin (lanes 5 and 6), nearly all of the α-actinin was recovered with F-actin in the pellet fraction following ultracentrifugation (lane 6). Following incubation and ultracentrifugation, bovine serum albumin (BSA) (negative control) revealed no interaction with F-actin and remained in the supernatant fraction (lane 7). (B) Wild-type and mutant ELMOD3 (Leu265Ser) co-sediment with polymerized F-actin. Lanes 1–2 and 5–6 contained the supernatant (S) and pellet (P) fractions of wild-type and mutant (p.Leu265Ser) TF-ELMOD3, respectively, which were obtained by ultracentrifugation. Both wild-type and mutant TF-ELMOD3 proteins were primarily recovered in the supernatant fraction (lanes 1 and 5). However, when incubated with polymerized F-actin (lanes 3 and 4), TF-ELMOD3 was present both in the supernatant (lane 3) and pellet fractions (lane 4), further confirming the association of ELMOD3 with F-actin. *In vitro*, the association of ELMOD3 with F-actin was unaffected by the Leu265Ser mutation in the ELMO domain (lanes 7 and 8).
(TIF)

Figure S10 *Elmod1-3* and *Arl2* are expressed in the mouse inner ear. RT-PCR of mouse cochlea and vestibular tissues from two different developmental stages revealed the expression of *Elmod1*, -*2*, -*3*, and *Arl2*. *Gapdh* was used as a control.
(TIF)

Table S1 Summary of exome sequencing analysis.
(DOCX)

Table S2 Primer sequences used to amplify and sequence *ELMOD3* coding and non-coding exons.
(DOCX)

Table S3 Predicted effect of p.Leu265Ser missense mutation on ELMOD3.
(DOCX)

Table S4 Taqman probes used for real time relative expression analysis of *ELMOD3/Elmod3* isoforms.
(DOCX)

Table S5 Primer sequences used for RT-PCR amplification.
(DOCX)
