# Supplementary figures and images for: An Alteration in ELMOD3, an Arl2 GTPase-Activating Protein, Is Associated with Hearing Impairment in Humans
Source: PLoS Genet. 2013 Sep 5;9(9):e1003774. doi: 10.1371/journal.pgen.1003774 (PMC3764207; doi:10.1371/journal.pgen.1003774)

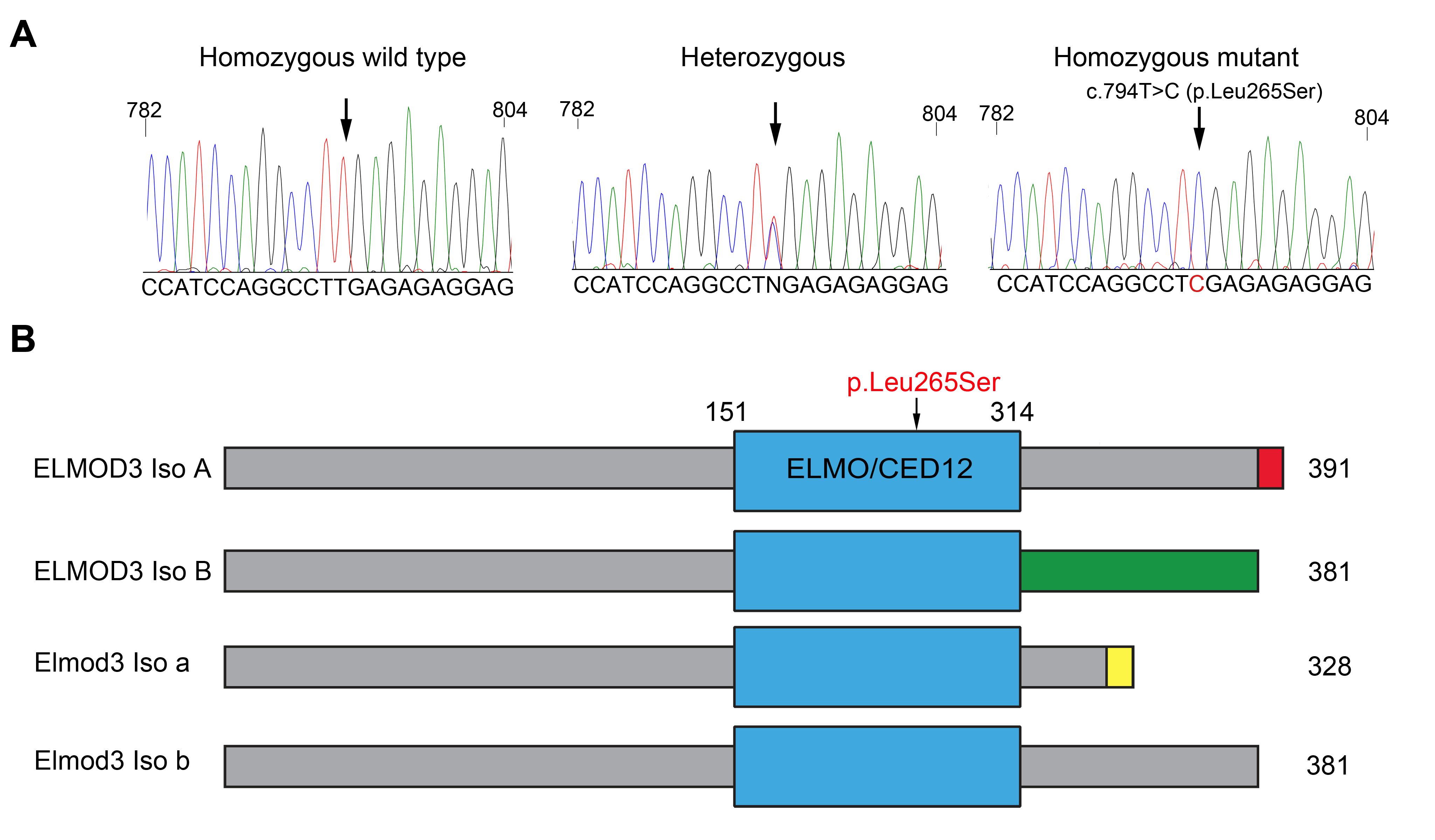

Supplement: Figure S1 — A missense mutation in the ELMO/CED12 domain of ELMOD3 is present in all the affected individuals of the PKDF468 family. (A) The nucleotide sequence chromatograms of exon 10 of ELMOD3, indicating the wild-type sequence, heterozygosity and homozygosity of the c.794T>C mutation. Nucleotide cDNA positions are given according to human ELMOD3 isoform A (accession no. NM_032213.4). (B) The predicted protein products of the different ELMOD3 splice variants. Both the A and B ELMOD3 isoforms contain an ELMO/CED12 domain and differ only at the carboxy termini. Mouse Elmod3 isoforms a and b are 79% and 82% identical to human ELMOD3 isoforms A and B, respectively. (TIF) [file pgen.1003774.s001.tif]

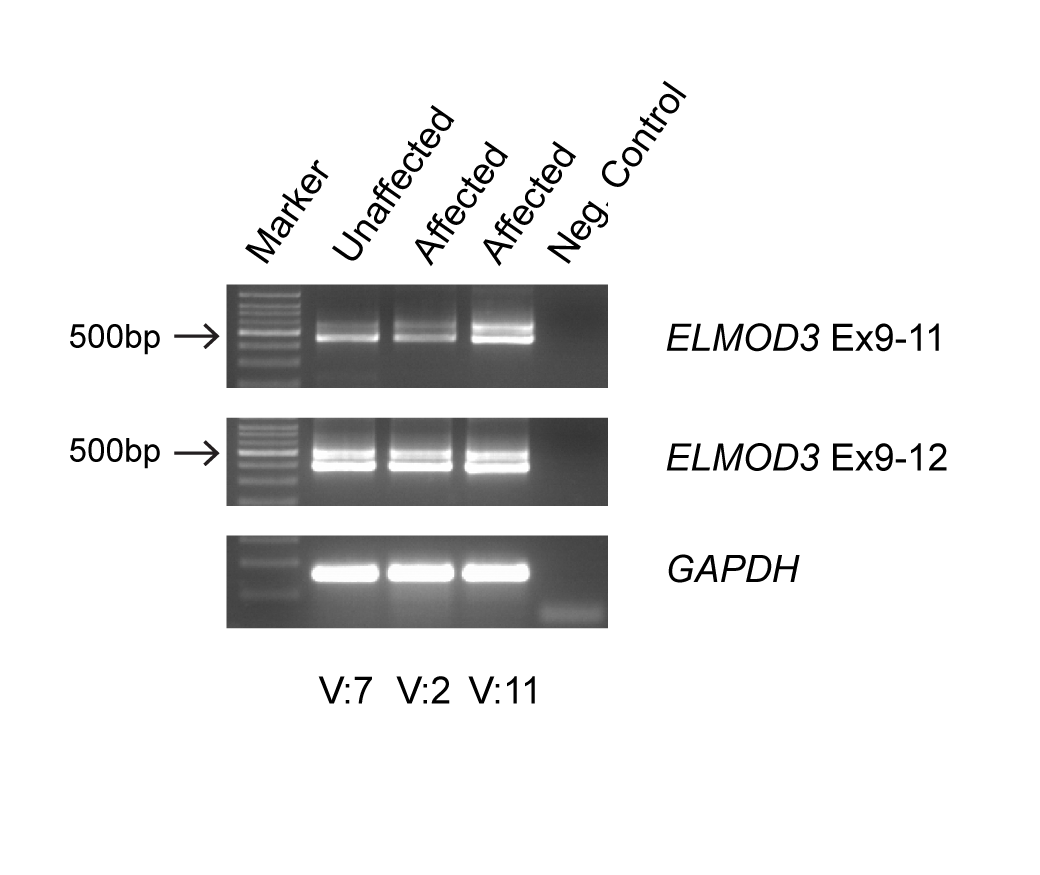

Supplement: Figure S2 — The c.794T>C mutation does not affect the splicing of ELMOD3 isoforms. Amplified ELMOD3 short transcripts from the cDNA libraries generated using the blood samples of affected and normal hearing individuals. GAPDH has been used as a positive control. (TIF) [file pgen.1003774.s002.tif]

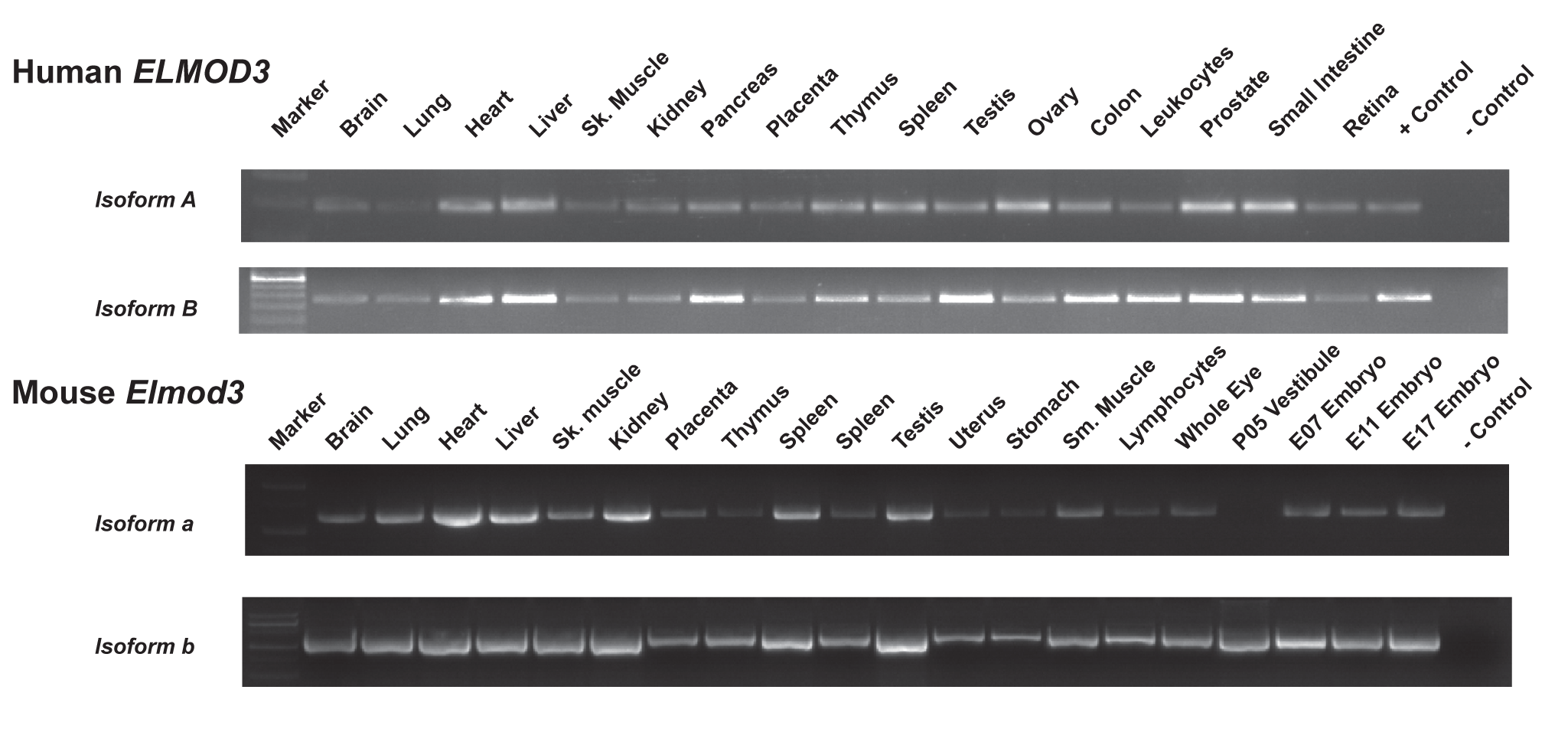

Supplement: Figure S3 — RT-PCR has been performed with human and mouse cDNA libraries. Human and mouse ELMOD3/Elmod3 isoforms A/a and B–D/b–c are expressed in many tissues. (TIF) [file pgen.1003774.s003.tif]

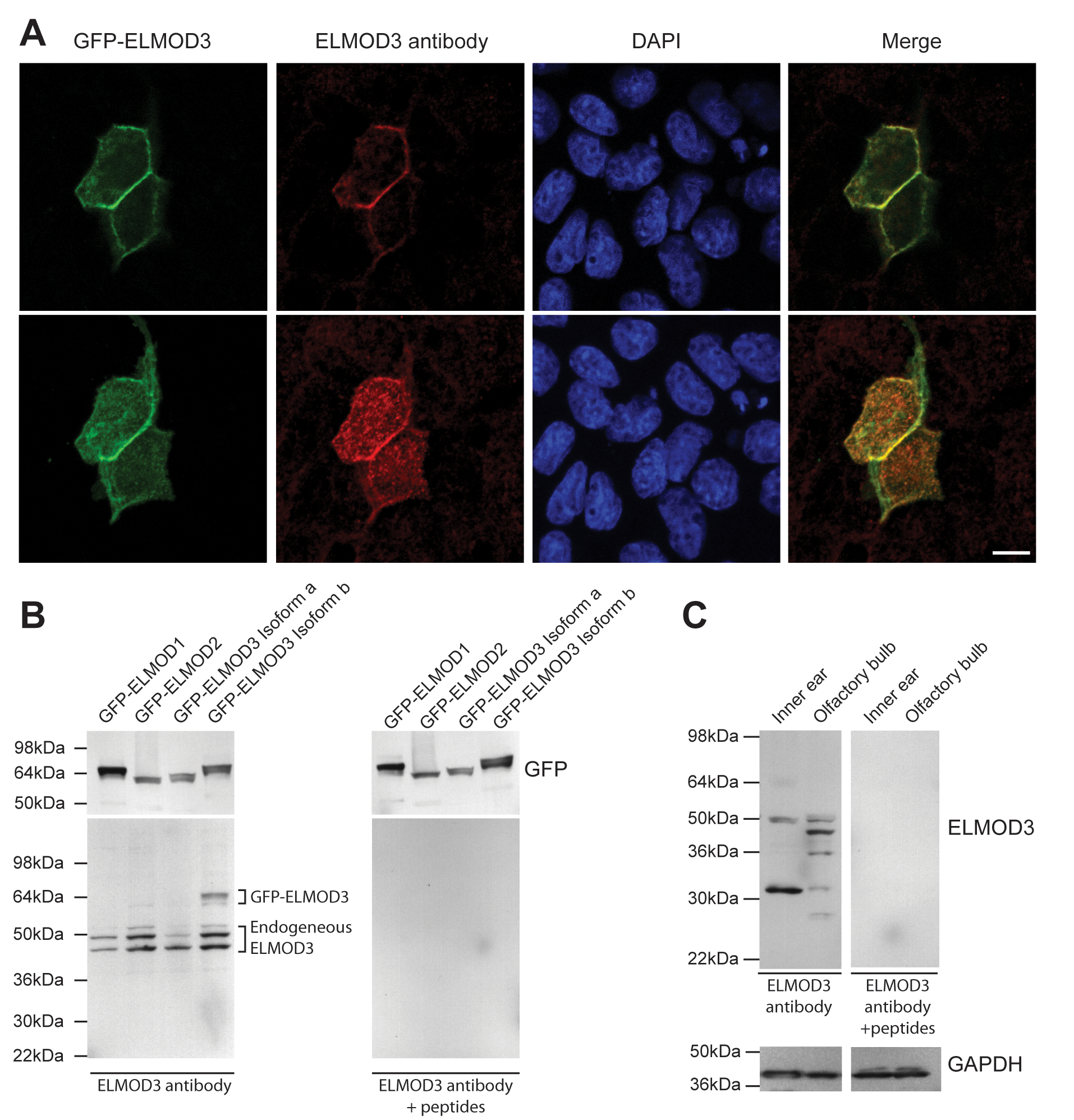

Supplement: Figure S4 — Our custom-made antibody against mouse recognizes mouse ELMOD3 isoform b. (A) To validate our custom-made antibody against mouse ELMOD3 in immunofluorescence, we performed a co-localization using full-length EGFP-tagged murine ELMOD3 isoform b transfected in MDCK cells. EGFP-ELMOD3 (green) staining and ELMOD3 antibody (red) staining colocalize (merge). Nuclei of transfected and non transfected cells have been counterstained with DAPI. The top panels represent a single confocal microscopy section of the cells at the nuclei level and the bottom panels a projection of all the confocal microscopy sections from the base to the apex of the cells. Scale bar: 10 µm. (B) Full-length EGFP-tagged murine Elmod1, Elmod2 and Elmod3 isoforms a and b cDNA constructs were expressed in HEK cells and Western blot analysis was performed on whole cell lysates. The GFP antibody was used as a loading and transfection control. The ELMOD3 antibody specifically recognized only the ELMOD3 isoform b and not the other known members of the ELMOD family. (C) Our custom-made antibody against mouse ELMOD3 recognizes endogenous protein from mouse tissues extracts (inner ear, olfactory bulbs). GAPDH has been used as a loading control. (TIF) [file pgen.1003774.s004.tif]

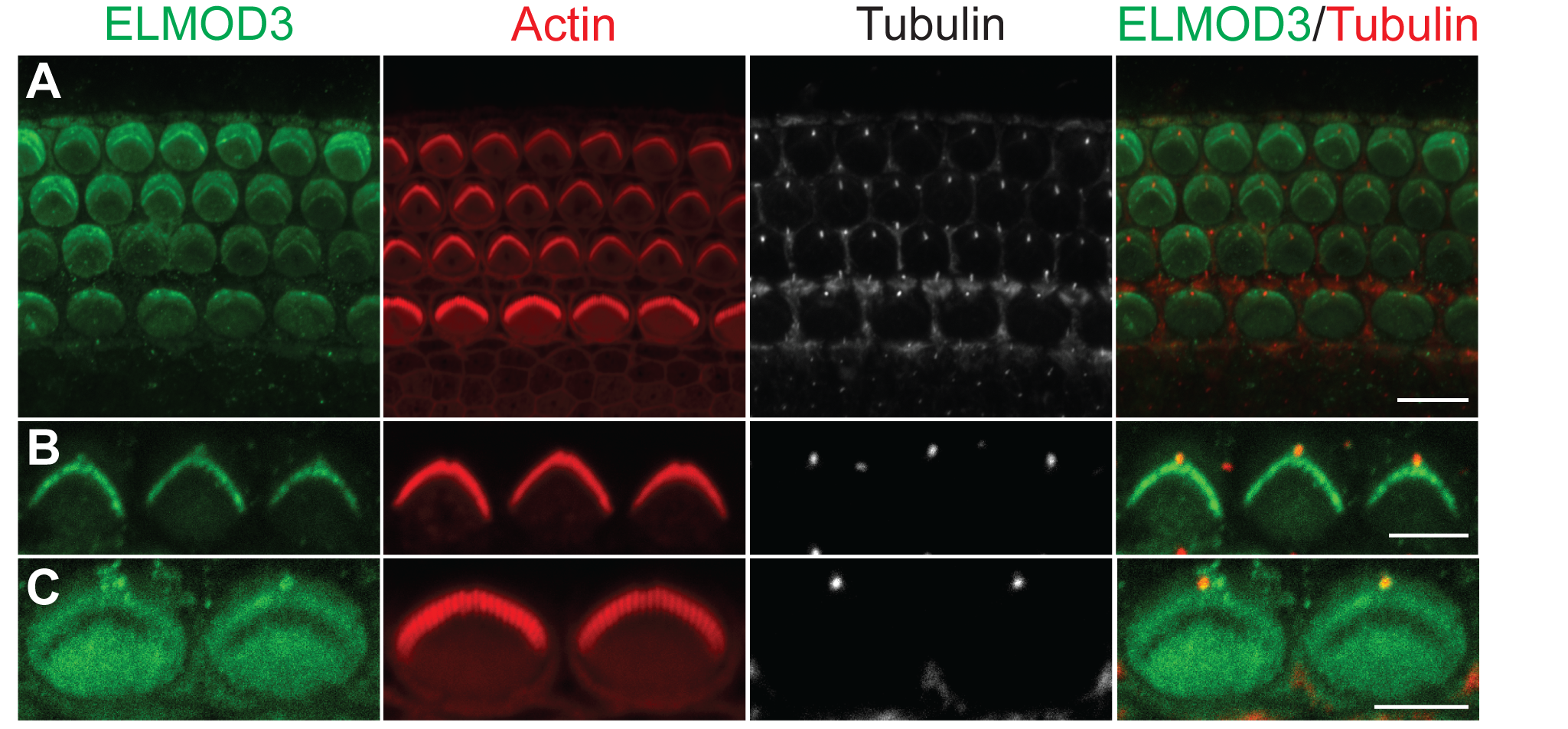

Supplement: Figure S5 — ELMOD3 immunoreactivity is localized in the kinocilia of developing auditory hair cells. (A) Maximal projection of confocal optical sections stacks of rat organ of Corti, at P02. ELMOD3 was immunostained in green while kinocilia were highlighted with acetylated-tubulin antibody (red). Actin cytoskeleton was labelled with rhodamine phalloidin (gray). (B) Single plane confocal acquisition of outer hair cells at the kinocilia level. (C) Single plane confocal acquisition of inner hair cells at the kinocilia level. ELMOD3 (green) immunoreactivity was found in the kinocilia (red) of both OHC and IHC in the organ of Corti of rat, at P2. Scale bar: 10 µm. Scale bars are 5 µm for higher magnification panels of IHC and OHC. (TIF) [file pgen.1003774.s005.tif]

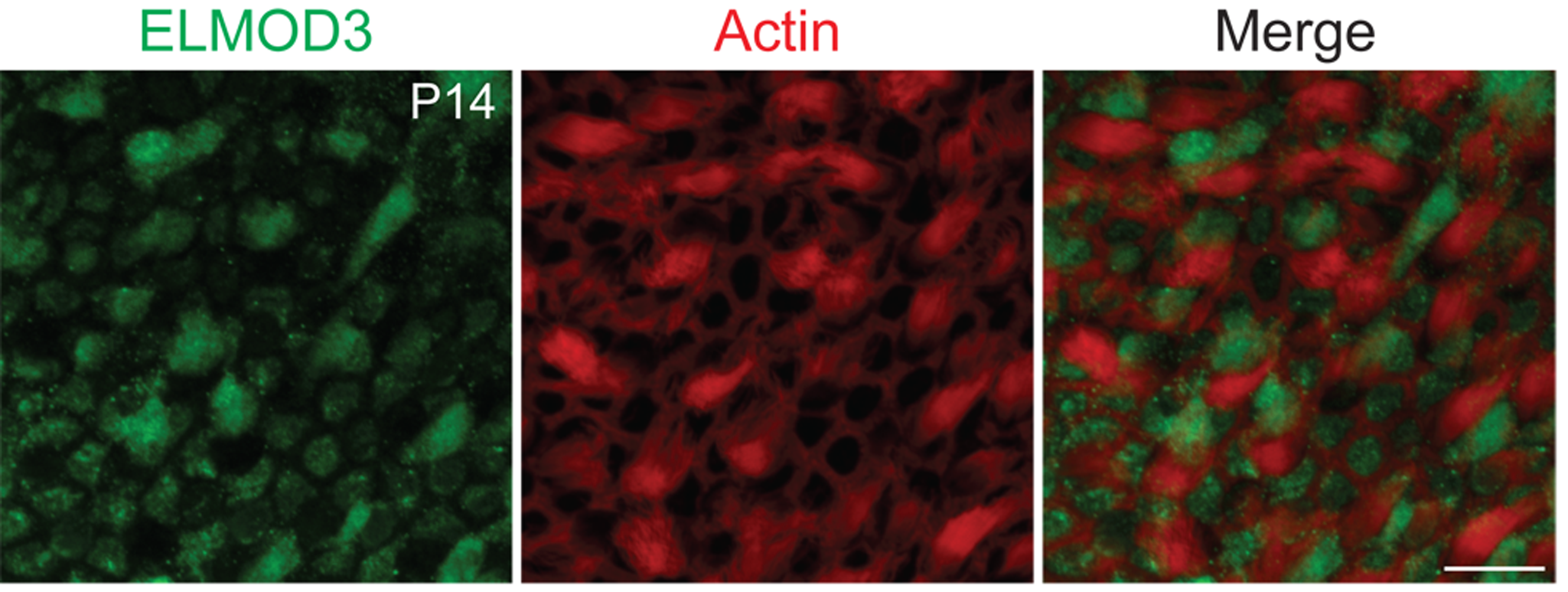

Supplement: Figure S6 — ELMOD3 immunoreactivity (green) was found within the hair cell and supporting cell bodies in the ampulla of rat, at P14, but no immunoreactivity was observed in the vestibular hair bundles. Actin cytoskeleton was highlighted with rhodamine phalloidin (red). Scale bar: 10 µm. All images are projection of confocal optical sections stack. (TIF) [file pgen.1003774.s006.tif]

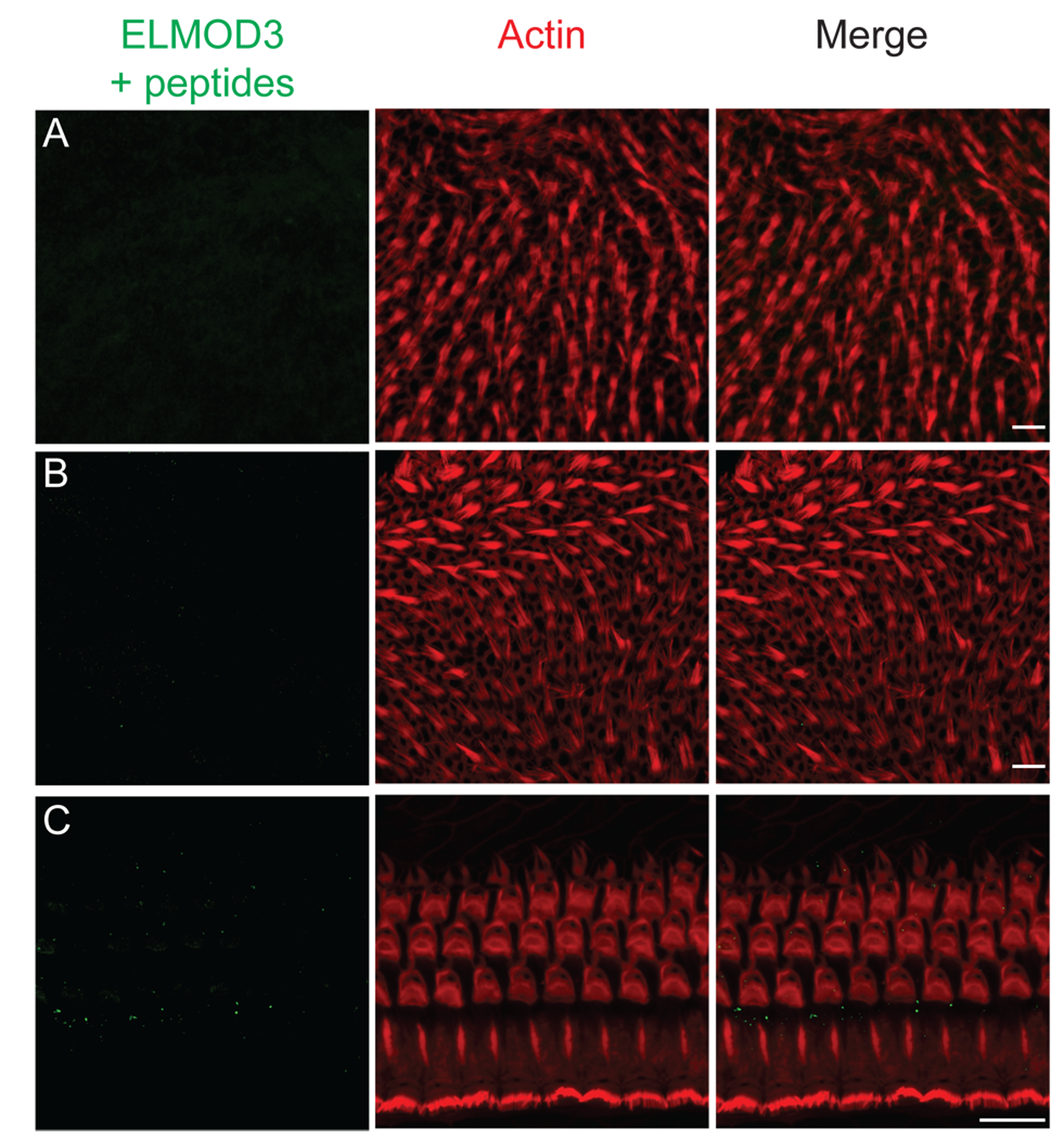

Supplement: Figure S7 — To validate our custom-made antibody against murine ELMOD3 in mouse tissues, we have blocked the antibody with the immunizing peptides followed by immunostaining on mouse inner ear epithelia at P14. Under these conditions, no staining has been observed in utricule (A), saccule (B) or the organ of Corti (C) epithelia. Scale bar: 10 µm. All images are projections of confocal optical sections stacks. (TIF) [file pgen.1003774.s007.tif]

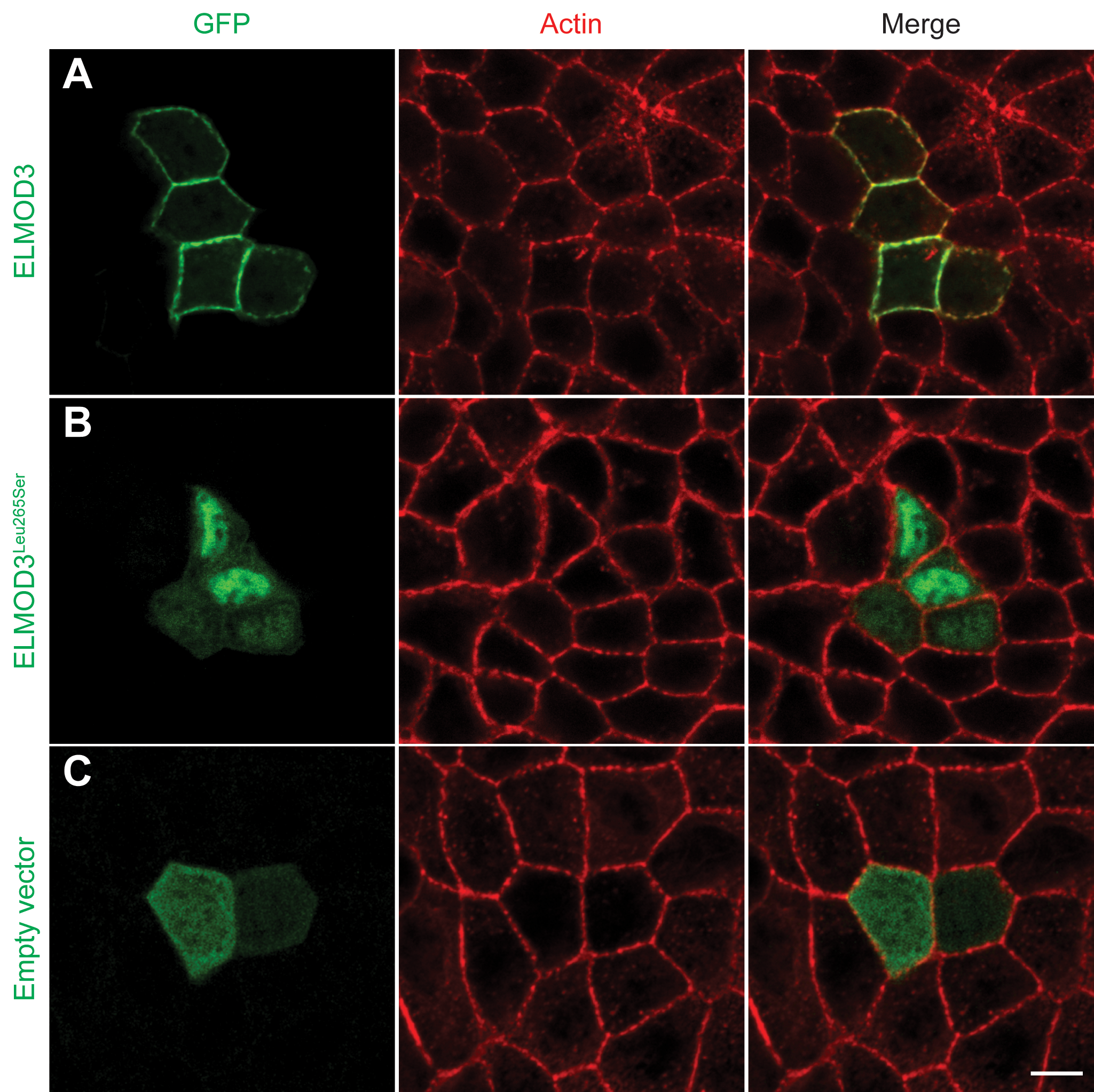

Supplement: Figure S8 — ELMOD3 targeting and localization with F-actin is affected by the DFNB88 allele. (A–B) The p.Leu265Ser mutation affected the targeting and localization of ELMOD3 to the actin cytoskeleton, stained with rhodamine phalloidin (red), at the cell membrane of MDCK cells. (C) pEGFP-C2 empty vector, which was used as a control. All the panels are confocal microscopy sections at the nuclei level. Scale bar: 10 µm. (TIF) [file pgen.1003774.s008.tif]

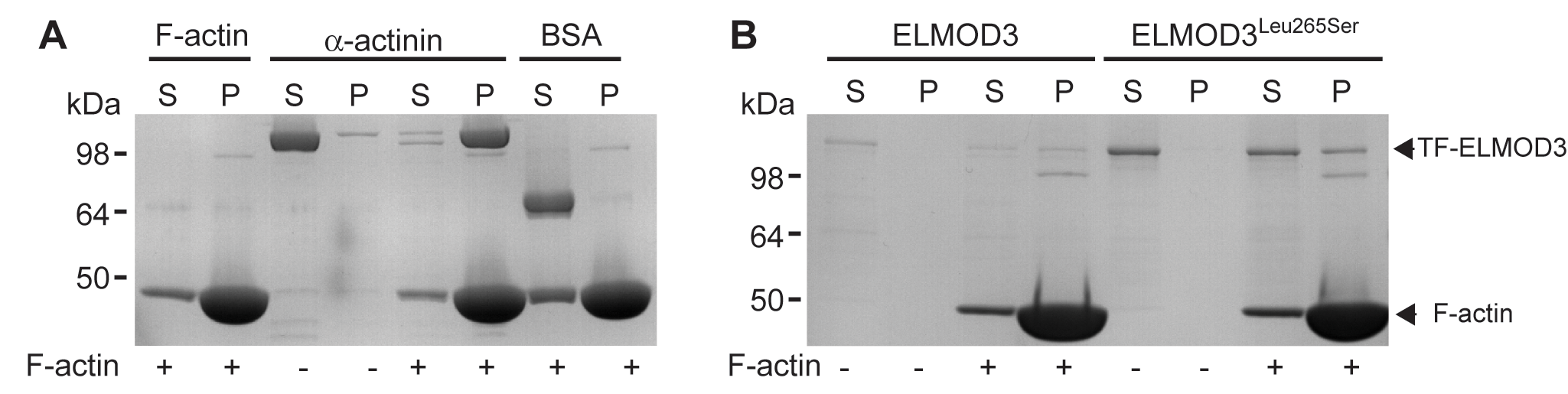

Supplement: Figure S9 — (A) Positive and negative controls for the F-actin co-sedimentation assay. The first two lanes contain the supernatant (S) and pellet (P) fractions of F-actin alone, respectively, which were obtained using high-speed centrifugation. F-actin was primarily recovered in the pellet fraction (lane 2). As a positive control, we used α-actinin, which is known to bind to F-actin. Following ultracentrifugation, α-actinin was recovered in the supernatant fraction (lane 3). When the same amount of α-actinin was incubated with F-actin (lanes 5 and 6), nearly all of the α-actinin was recovered with F-actin in the pellet fraction following ultracentrifugation (lane 6). Following incubation and ultracentrifugation, bovine serum albumin (BSA) (negative control) revealed no interaction with F-actin and remained in the supernatant fraction (lane 7). (B) Wild-type and mutant ELMOD3 (Leu265Ser) co-sediment with polymerized F-actin. Lanes 1–2 and 5–6 contained the supernatant (S) and pellet (P) fractions of wild-type and mutant (p.Leu265Ser) TF-ELMOD3, respectively, which were obtained by ultracentrifugation. Both wild-type and mutant TF-ELMOD3 proteins were primarily recovered in the supernatant fraction (lanes 1 and 5). However, when incubated with polymerized F-actin (lanes 3 and 4), TF-ELMOD3 was present both in the supernatant (lane 3) and pellet fractions (lane 4), further confirming the association of ELMOD3 with F-actin. In vitro, the association of ELMOD3 with F-actin was unaffected by the Leu265Ser mutation in the ELMO domain (lanes 7 and 8). (TIF) [file pgen.1003774.s009.tif]

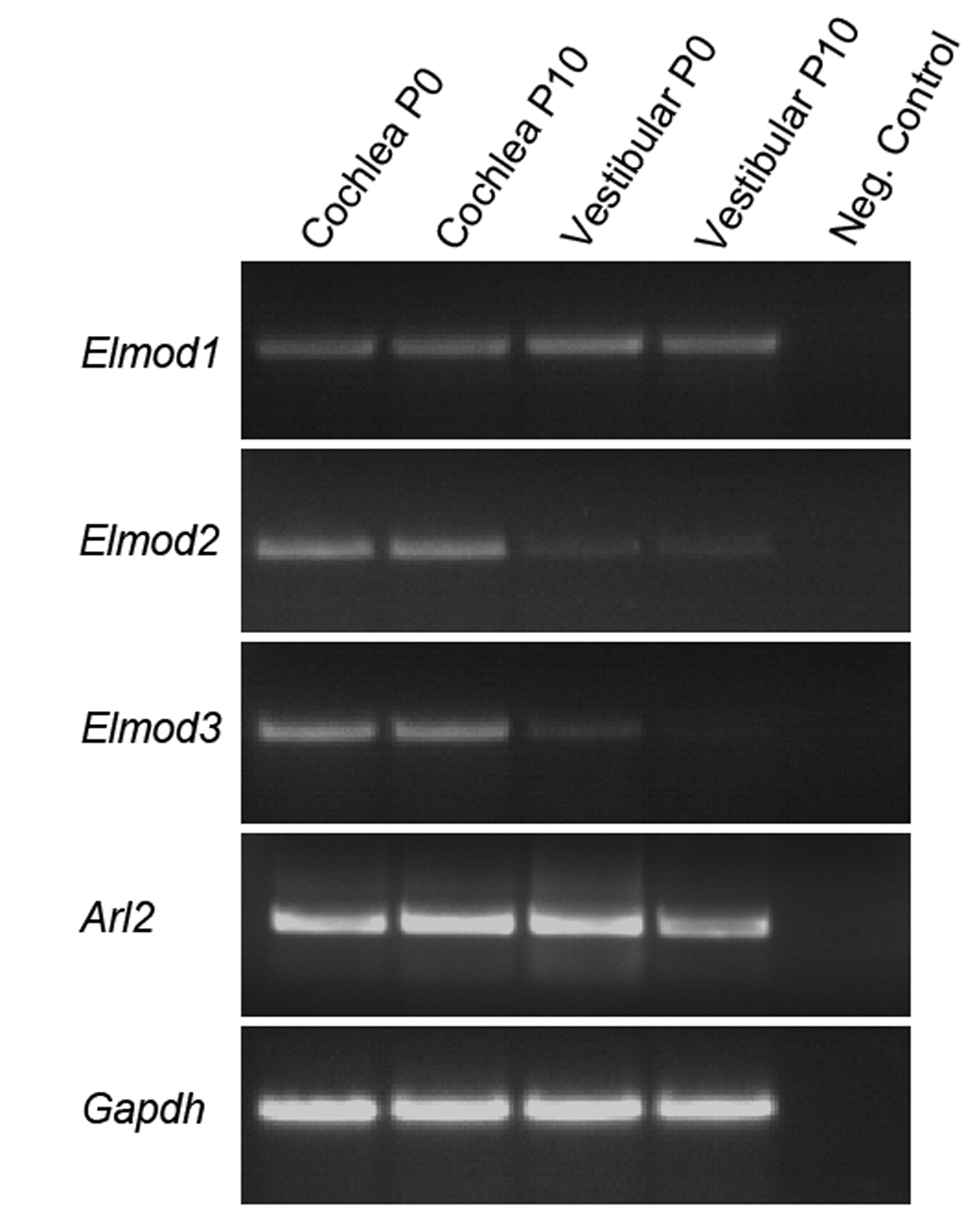

Supplement: Figure S10 — Elmod1-3 and Arl2 are expressed in the mouse inner ear. RT-PCR of mouse cochlea and vestibular tissues from two different developmental stages revealed the expression of Elmod1, -2, -3, and Arl2. Gapdh was used as a control. (TIF) [file pgen.1003774.s010.tif]
