# Supplementary material for: An Alteration in ELMOD3, an Arl2 GTPase-Activating Protein, Is Associated with Hearing Impairment in Humans
Source: PLoS Genet. 2013 Sep 5;9(9):e1003774. doi: 10.1371/journal.pgen.1003774 (PMC3764207; doi:10.1371/journal.pgen.1003774)
Supplement: Table S1 — Summary of exome sequencing analysis. (DOCX) [file pgen.1003774.s011.docx]

**Table S1:** Summary of Exome sequencing analysis

|  | **Exome** | ***DFNB88* locus** |
| --- | --- | --- |
| Total changes  Changes not in dbSNP135 (Minor allele frequency ≥0.05)  Non-synonymous/Splice site/insertions/deletions  Genes with homozygous/compound heterozygous changes  Changes not found in Pakistani control samples  Changes not found in 1000 genome or NHLBI ESP  Potential pathogenic changes in known deafness genes  Changes predicted to be pathogenic^#^  Changes segregating with hearing loss in family PKDF468 | 64,863  1,928  312  30  30  17  0  8  1 | 27  1  1  1  1  1  0  1  1 |

^#^Pathogenic predicted by at least two of the four prediction programs: Polyphen-2, SNPs3D, MutationTaster and SIFT.
