# Supplementary material for: An Alteration in ELMOD3, an Arl2 GTPase-Activating Protein, Is Associated with Hearing Impairment in Humans
Source: PLoS Genet. 2013 Sep 5;9(9):e1003774. doi: 10.1371/journal.pgen.1003774 (PMC3764207; doi:10.1371/journal.pgen.1003774)
Supplement: Table S2 — Primer sequences used to amplify and sequence ELMOD3 coding and non-coding exons. (DOCX) [file pgen.1003774.s012.docx]

**Table S2:** Primer sequences used to amplify and sequence *ELMOD3* coding and non-coding exons.

| **Exon** | **Forward Primer** | **Reverse Primer** | **Product (bp)^a^** |
| --- | --- | --- | --- |
| 1a | AGCGAAGAGCCAACTCTCAG | AGTCGGGATTCTCTTTCAGTCTTAC | 343 |
| 1b | TGAGGAGGGAGGGAGAATGT | ATTTAAATTAGATGGGCCCTTAGC | 459 |
| 1 | CCTAAAGAGGGTGCATAATGAATAC | GGAAGAATACCTCCTGGCTCTT | 497 |
| 2 | ATCCCTTCTAGTGGTGCTGCTG | ATCTCGGCTCACTGCAACCT | 400 |
| 3 | CACAAAGGGACTGTTTTCTTACTCT | CAAAACAAGCTATTAGGAAAAGCAA | 400 |
| 4 | CAAATACCTGGCTGATGAAATAAC | CTCACTCCCCTCAAGTGTGA | 400 |
| 5 | TAAAACTGACTTCAGTCAACCCATC | CTCTGGTCTTTTGATTCCTAATTCA | 393 |
| 6 | ACAGTGACACCCCATCTCTTAAA | ATAGTTAAAATGAGCACTCTCAGGA | 323 |
| 7 | TCTTGAAAGAAGATTGGTTGTAGC | CAAATGAAGCATGGGTCCTA | 397 |
| 8 | CTAGAGACTCAACAGGTGCCTTG | TCTCAGAGAATGTGAGATCTGGACT | 462 |
| 9 | AATATTGCATGTACCATGGTTGG | CACCCTCTTCCCGGAATACT | 400 |
| 10 | CATTAGAATGAGCCCCACAAGT | GGTACAGCTCATATTTCAATGCAG | 431 |
| 10a | CACTGAGCCTTAGTTCCTCATCTGT | ATTCCCCAGGAGCCTGACAT | 452 |
| 11 | ATGTCAGGCTCCTGGGGAAT | TGGTAAGCTGGAATTTGAGAAAGTG | 400 |
| 12 | CTAGGTCTCAGCCTTCATCCAC | CCACAGGGACCACACACTCTA | 500 |
| 13 | ATCTGAGGCTCCTTCACTTTCTCT | GAAGGGGGTGAAGTCTAGCTG | 538 |

^a^All PCR products were amplified with 1.5 mM MgCl_2_ and 60^o^C annealing temperature, except primers for exons 1, 10a and 11 that required 63^o^C.
