# Supplementary material for: An Alteration in ELMOD3, an Arl2 GTPase-Activating Protein, Is Associated with Hearing Impairment in Humans
Source: PLoS Genet. 2013 Sep 5;9(9):e1003774. doi: 10.1371/journal.pgen.1003774 (PMC3764207; doi:10.1371/journal.pgen.1003774)
Supplement: Table S3 — Predicted effect of p.Leu265Ser missense mutation on ELMOD3. (DOCX) [file pgen.1003774.s013.docx]

**Table S3:** Predicted effect of p.Leu265Ser missense mutation on ELMOD3

| **Program** | **Predicted Effect (score)** |
| --- | --- |
| PolyPhen-2* | Probably damaging (0.991) |
| SNP3D | Damaging (-0.17) |
| Mutation Taster^#^ | Disease causing (3.95) |
| PMut^$^ | Pathological (reliability 5) |
| SIFT | Damaging |

*****Score range from 0 (polymorphism) to 1.0 (possibly damaging)

**^#^**Score range from 0 (polymorphism) to 6.0 (disease causing)

^$^Reliability value range from 0 (low reliable) to 9 (very reliable).
