# Supplementary material for: An Alteration in ELMOD3, an Arl2 GTPase-Activating Protein, Is Associated with Hearing Impairment in Humans
Source: PLoS Genet. 2013 Sep 5;9(9):e1003774. doi: 10.1371/journal.pgen.1003774 (PMC3764207; doi:10.1371/journal.pgen.1003774)
Supplement: Table S4 — Taqman probes used for real time relative expression analysis of ELMOD3/Elmod3 isoforms. (DOCX) [file pgen.1003774.s014.docx]

**Table S4:** Taqman probes used for Real Time relative expression analysis of *ELMOD3*/*Elmod3* isoforms

| **Target** | **Forward Primer** | **Probe** | **Reverse Primer** |
| --- | --- | --- | --- |
| *Isoform A* | GTGTCTCTCCAGAGAGTGAAT | CTTCTATGCCGCCACATTCCTCCA | GAAGAAAGAGCACACCTTTGA |
| *Isoforms B-D* | ATCTCAGACTCGGGCTTTGT | AGTATTGGCCAAGAAGAGCCCACG | TTGACACCCTGGCCAAGTA |
| *GAPDH* | TGTAGTTGAGGTCAATGAAGG | AAGGTCGGAGTCAACGGATTT | ACATCGCTCAGACACCATG |
| *Isoform a* | AACATCACCCGCATTGCCATCCA | ACAGCTTCTACGCGGCTACTTTCCTT | TGGACACAGGAGGAAGTGCC |
| *Isoforms b-c* | TGATGTCTGTGAACATCACCCGCA | AGACTTGGAAGCGTTGGCCAAGAAGA | ATCCAGGGTCTTGAGCAGTCGTTT |
| *Gapdh* | AATGGTGAAGGTCGGTGTG | TGCAAATGGCAGCCTGGTG | GTGGAGTCATACTGGAACATGTAG |
