# Supplementary material for: An Alteration in ELMOD3, an Arl2 GTPase-Activating Protein, Is Associated with Hearing Impairment in Humans
Source: PLoS Genet. 2013 Sep 5;9(9):e1003774. doi: 10.1371/journal.pgen.1003774 (PMC3764207; doi:10.1371/journal.pgen.1003774)
Supplement: Table S5 — Primer sequences used for RT-PCR amplification. (DOCX) [file pgen.1003774.s015.docx]

**Table S5:** Primer sequences used for RT-PCR amplification

| **Gene** | **Forward Primer** | **Reverse Primer** | **Product (bp)** |
| --- | --- | --- | --- |
| *hELMOD3_IsoA* | GTGAACATCACCCACATTGC | GAAAGTGTGGATGGGGAAGA | 295 |
| *hELMOD3_IsoB-D* | CAATTCCCTTTCTGTTTGATGTC | ACCTGTGAAGGTGAGATCCTTG | 360 |
| *hELMOD3*_*IsoA* full length | CGAgaattcATGAATGAAAAATCTTGCTCTTTCCATAG | CGAggatccACAGGGACCACACACTCTACCTTGT | 1258 |
| *hELMOD3*_*IsoB* full length | CGAgaattcATGAATGAAAAATCTTGCTCTTTCCATAG | CGAggatccTCAGCCTTTAAGCCTCCATTCATC | 1177 |
| *hELMOD3*_*ex9-11* | TACCTGGTGATGGACTCAAAGAC | CAGGGCAGAGAAAGGAGAACTA | 462 590 |
| *hELMOD3*_*ex9-12* | TACCTGGTGATGGACTCAAAGAC | CTTCTGGCCCATAGCACTTCT | 400 528 |
| *mElmod3_ Iso a* | CCTTCTGTTTGATGTCTGTGAACAT | CCTGGACACAGGAGGAAGTG | 233 |
| *mElmod3*_*Iso b-c* | CCTTCTGTTTGATGTCTGTGAACAT | CCTCCAACAAGGAGGTCTGTC | 297 |
| *mElmod3* | ACACCATCCATCCAGACATAGAG | TTGAGTCCATGACCAGGTAGAGTAG | 438 |
| *mElmod1* | TGAAACGTCACTGAGAGATTCAAA | TATTGCAAATTGTAAAGTCCCAGAA | 396 |
| *mElmod2* | TTCCAAGAATAAGGTCCTACAGAAT | TTCCAACTATTGCATAAGAATACCC | 467 |
| *mGapdh* | TGATGACAAGAAGGTGGTGAAG | TCCTTGGAGGCCATGTAGGCCAT | 230 |
